# How the Identity of Substance Users Shapes Public Opinion on Opioid Policy

**DOI:** 10.1007/s11109-022-09845-8

**Published:** 2022-12-20

**Authors:** Justin de Benedictis-Kessner, Michael Hankinson

**Affiliations:** 1 John F. Kennedy School of Government, Harvard University, 79 John F. Kennedy St., Cambridge, MA 02138 USA; 2 Department of Political Science, George Washington University, 2115 G Street, N.W., Washington, DC 20052 USA

**Keywords:** Group identity, Race, Public health, Addiction, Healthcare, Public policy

## Abstract

How do media portrayals of potential policy beneficiaries’ identities sway public support for these policies in a public health setting? Using a pre-registered vignette experiment, we show that the racial identity of substance users depicted in news media shapes public opinion on policies to address the opioid crisis. People display biases in favor of their own racial identity group that manifest in their support for both treatment-based policies and punitive policies. We show that these biases may be moderated by the type of initial drug used by a substance user and associated levels of perceived blame. Extending theories of group politics, we also assess favoritism based on gender and residential context identities, but find no such biases. These results highlight the continued centrality of race in the formation of policy preferences.

**Supplementary Information:**

The online version contains supplementary material available at 10.1007/s11109-022-09845-8.

Each day, more than 200 Americans die from a drug overdose, making overdose the leading cause of death for Americans under age 45 (Woolf et al., 2021). The rise in overdoses has been driven by the use of opioids, and in particular synthetic opioids such as fentanyl: nearly three-quarters of overdoses in 2020 involved opioids (Baumgartner & Radley, 2021). Though opioid overdoses are not a new phenomenon, the current opioid crisis has permeated the media with stories of substance use and addiction, thereby raising awareness of these issues among the public. This has been accompanied by increased calls for healthcare policies—specifically, addiction treatment programs—suited to address the crisis (Om, 2018; Saloner & Barry, 2018). In contrast, legislative responses to previous drug crises were more punishment-oriented (Kim et al., 2020).

What has caused this increased attention by the media, the public, and policymakers to drug addiction, and a focus on treatment-based rather than punitive policy responses? Popular narratives have focused on how, compared to previous drug addiction crises, the current opioid crisis affects different people and places. Specifically, the current opioid crisis cuts across lines of class, race, and ideology, and reaches more rural, whiter, conservative, and less wealthy parts of the United States (Jalal et al., 2018). In contrast, previous drug crises—such as the crack epidemic of the 1980s—affected largely non-white populations living in urban population centers. In turn, substance users depicted in media stories on the current opioid epidemic have been whiter and less urban than in media narratives during the crack scare (Harbin, 2018; Netherland & Hansen, 2016).

These differences in the identities of opioid substance users may be what has caused public opinion and subsequently public policy to support treatment rather than punishment much more than during past drug crises. Past research has shown that associating racial minorities with other policies—from welfare, to the Affordable Care Act (ACA), to gun ownership—may cause white Americans to oppose those policies (e.g., Gilens, 1999; Hayes et al., 2020; Tesler, 2012). The whiter media narrative surrounding the opioid crisis could, in an opposite effect, drive members of the public to support compassionate opioid treatment policies. Testing the way that multiple group identities influence policy opinions is crucial for understanding both how policymakers and the public have responded to the opioid crisis. Such tests can also help assess the political feasibility of future policy solutions related to both opioids and other contemporary health crises.

In this paper, we empirically assess how the identity of substance users depicted in the media shapes public opinion on policy responses to the opioid crisis using an experiment. We compare people’s responses to media descriptions of people with substance use disorder in the current drug crisis using a pre-registered factorial randomized survey experiment that varies features of potential policy beneficiaries.^1^ We manipulate the racial identity, gender, and residential location of opioid users depicted in a media story to examine how these identities shape support for both treatment-based and punitive policy. Moreover, we explore one mechanism behind these effects on policy opinions advanced by other theories of social policy: the perceptions of individual blame for addiction.

Our results highlight the continued primacy of race in shaping public support for targeted social policies. We show that people increase their support for treatment-based policy when they are shown stories of substance users who share their own racial identity. We test for whether group identities beyond race—gender and geography, two increasingly salient political cleavages in American politics—have the potential to also influence the public’s support for policy. The effects of these other group identities pale in comparison to the effect of race, suggesting that public opinion surrounding the opioid crisis is highly racialized but likely not shaped by other group identities in ways suggested by some popular media accounts.

Additionally, we test a commonly believed mechanism for the relationship between group identity and resource allocation: the perceived deservingness of policy recipients. Although we find that blame is strongly correlated with treatment policy support, we do not find evidence that blame mediates the relationship between group identity and policy support. We do, however, find that a person’s initial use of different types of opioids—heroin rather than prescription pills—directly affects perceptions of blame and conditions the relationship between shared identity and support for opioid policy. This suggests that blame can moderate the role of group identity in the formation of policy opinions, even if it does not causally mediate this relationship.

Together, these results demonstrate how media depictions of the people affected can shift public opinion about policies to address the opioid crisis. Although our findings cannot speak to the cumulative effects of exposure to multiple media stories, our findings still present a normatively troubling possibility for policy representation, especially for the nuanced issue of health policy. The portrayal of the opioid crisis as predominantly affecting white populations may have increased policy support among white constituents and policymakers. Yet our findings also suggest that health crises disproportionately affecting communities of color—such as COVID-19—may be less likely to receive similar support for compassionate medical policy if the media portrays them as accurately having such disproportionate impacts.

## Theory and Hypotheses

Media stories about the opioid crisis have differed in their coverage from those during previous drug crises. Coverage has often featured white rather than non-white substance users (Harbin, 2018), as well as people from backgrounds outside of urban environments (Netherland & Hansen, 2016). The language in such media portrayals has also highlighted medical policy responses rather than criminal justice policy responses to the crisis (Shachar et al., 2020). The racial identities of the people and the language used in these stories may be one reason support for opioid treatment policies is so high (de Benedictis-Kessner & Hankinson, 2019). Specifically, both popular media and research studies have claimed that ‘whiteness’ is driving national attention to the current epidemic (Netherland & Hansen, 2016). *These claims lead us to expect that our nationally-representative sample on the whole will be less supportive of funding treatment programs after reading about a Black rather than a white policy beneficiary (H1).*

Though previous research has noted that the sympathetic framing of individuals in news articles about the opioid crisis can shape public opinion about policy responses (Raychaudhuri et al., 2022), it has been unable to determine the role of *shared* identity, nor has it been able to explore the role of identity traits other than race. Our study explores group attachments and identity beyond the effect of race on white Americans. While a large body of research suggests that racial group identity is influential in the formation of people’s policy preferences, other (often intersecting) group identities of people may also shape attitudes. We test a theory that incorporates the potential for multiple types of group identity—both relatively immutable group characteristics and group traits less commonly thought of as structuring social preferences—to shape policy preferences.

Within the United States, racial identity often provides a foundational group attachment that structures policy attitudes. For example, when white Americans believe that policies will target benefits to Black people—not necessarily in an accurate reflection of reality—they are often less supportive of these policies than when they believe these policies will benefit white people (e.g., Feldman & Huddy, 2005; Gilens, 1999). These dynamics play out in numerous policy areas, including welfare policy, affirmative action, crime, and taxes. Health care policy opinions in particular have been recently shaped by racial bias (Israel-Trummel & Shortle, 2019; Tesler, 2012). Black Americans also express support for social programs benefiting ingroup members, though this support may weaken when the issue is linked to a marginalized subset of the ingroup (White, 2007). This literature leads us to expect that *white respondents will report higher levels of support for opioid treatment funding when white rather than Black substance users are depicted in the media, while Black respondents will report higher support when Black rather than white substance users are depicted (H2a).*

The group attachments that influence support for public policies may be especially broad in the case of policies to address opioid use given the unusual context of the opioid crisis. This context makes this issue area an excellent place to test how group identities other than race shape attitudes. For example, while heroin and non-medical prescription opioid use is greater among men, the rate of use is growing faster among women (Marsh et al., 2018), and pregnant women have historically been especially stigmatized for drug use (Gomberg, 1982). These gendered perceptions of drug use may strengthen the salience of gender identity in this issue area. More broadly, women politicians and members of the public tend to support social policies that target women (e.g., Holman, 2014; Strolovitch, 2008). *We therefore expect both men and women respondents will be more supportive of treatment funding when viewing substance users who match their gender (H2b).*

Especially unusual about the opioid crisis is its geographic context. Unlike past drug crises, the opioid crisis has been characterized by higher rates of prescription drug misuse and overdose in rural areas of the US than in urban areas (Monnat & Rigg, 2016). Americans may therefore have opinions on opioid-related policies associated with the rural or urban identities of the people these policies target (Lyons & Utych, 2021; Nemerever & Rogers, 2021). People may even display residential context-based ingroup preferences reflective of Cramer’s (2016) theory of ‘rural consciousness’ and the growing urban-rural political divide, despite geographic identity being more malleable than other identity characteristics. *We expect respondents will be more supportive of treatment funding when viewing substance users who share their residential context (H2c).*

Much of the literature on the role of group identity in shaping policy preferences has focused on *beneficial* policies that would confer positive benefits on people of certain identity groups. Other social policies do not directly benefit those who interact with them. Many policies proposed to address illicit drug use—such as the opioid crisis—involve more *punitive* policies. If the punitive policy is viewed as a threat to members of one’s ingroup, it could lead respondents to oppose the policy (Klar, 2013). Conversely, if the policy is perceived as helping to maintain ingroup norms by punishing deviant behavior (i.e., a “black sheep effect”), respondents may respond to a shared identity with the affected individual by being more supportive of punitive policies (Marques & Paez, 1994).

In particular, white Americans may be more supportive of punitive policies that will impact Black people due to outgroup biases (Hurwitz & Peffley, 2005b), and Black Americans may oppose punitive policies that target racial ingroup members. Historic racial bias in the enforcement and sentencing of drug offenses during the crack epidemic likely informs many Black Americans’ perceptions of narcotics law enforcement as unfair (Bobo & Johnson, 2004). This history could discourage support for law enforcement policy responses when the policy recipient is Black. In line with this, public opinion data show Black Americans are more willing to allocate resources to lowering crime rates than white Americans—likely due in part to their higher probability of being victims of crime—but also are more concerned over the harshness of police violence (Eckhouse, 2019). On the other hand, Black Americans could instead see punitive policies as a way to enforce group norms due to the dynamics of “respectability politics” (Forman, 2017; Jefferson, 2020). *Despite these contrasting theories, we expect both white and Black respondents to show less support for funding punitive policy when reading a profile of a substance user from their racial ingroup (H3a).*

The role of gender and geographic group identities in support for punitive policies has weaker theoretical and empirical foundation on which to build our theory. Shared gender and geographic context identities may simply shape support for law enforcement policy in the reverse of how they shape support for treatment policy. *We expect that people will be less supportive of punitive policy targeted towards people who share a gender identity (H3b) or geographic context identity with them (H3c).*

The mechanism behind ingroup biases in people’s drug policy preferences is not clearly informed by previous research. Group identity might shape opioid policy opinions through people’s perceptions of individual blame. Substance users have traditionally been viewed as personally responsible for addiction and thus undeserving of assistance (Jencks, 1992). Yet unlike in past drug crises wherein substance abusers were seen as deviant, many Americans view the opioid epidemic as an unclear case of personal responsibility. A 2017 poll found that 69% of Americans can “understand how someone accidentally gets addicted to opioids” (American Psychiatric Association, 2017). A likely reason for this is that many opioid addictions begin with painkillers prescribed by a doctor (Cicero et al., 2014).

Group identity may contribute to this social construction of deservingness (Fang & Huber, 2020; Schneider & Ingram, 1993; Tajfel, 1982). Racial identity may play a particularly potent role in the mechanism behind the formation of opinion on public policies and, in our case, opioid treatment policy (Michener, 2019).^2^ In the case of opioid-related policies, people’s support may therefore be shaped by group identity via their perceptions of a substance user’s blame for their own addiction. *We therefore expect people’s perceptions of blame to negatively correlate with their support for funding treatment policy and positively correlate with support for funding punitive policy (H4)*. In turn, blame may play a causally mediating role in policy attitudes (Imai et al., 2010, 2011). *We expect that blame will mediate people’s support for policies that are both beneficial and punitive (H5).*

## Research Design and Data

To test these hypotheses, we use a vignette-style factorial randomized survey experiment that allows us to vary information in a news article describing a person struggling with substance use. We constructed this news article by combining elements of text from actual published news articles that profiled individuals who use opioids.^3^ Crucially, we manipulated identity-based attributes of the substance user depicted in this story. Each story featured a substance user with a randomly chosen racial identity, gender, and residential location. We also varied the initial opioid drug they were described as first using before developing a substance use disorder, and the source of insurance for their addiction treatment.^4^ The research design, protocols, hypotheses, and analyses were pre-registered prior to data collection.

**Fig. 1:**
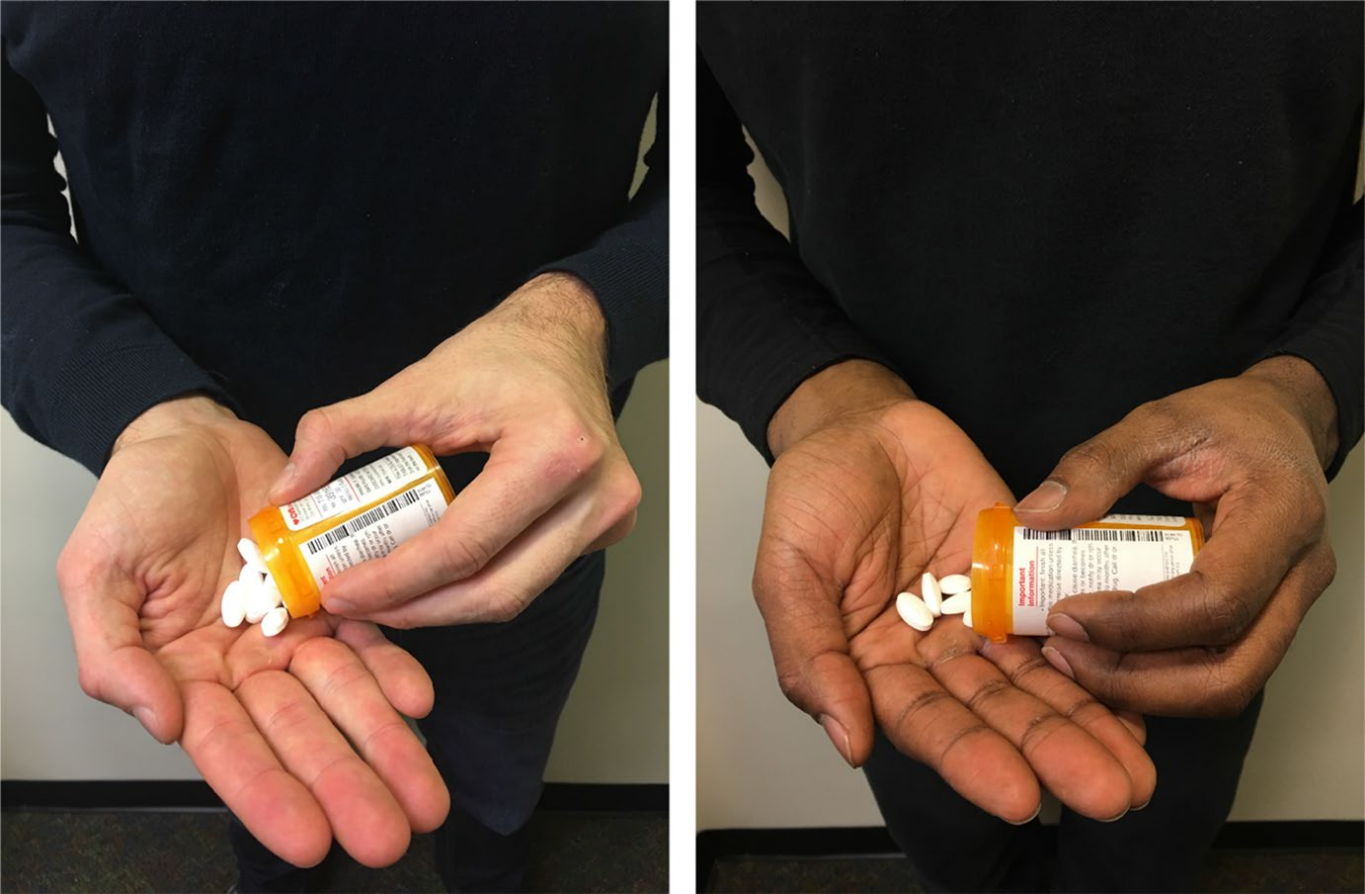
Example photos accompanying news story (from the white/Black male Oxycontin conditions)

We varied the racial identity of the policy recipient described in the article to be either non-Hispanic white or African-American.^5^ We vary racial identity by both presenting different photos at the beginning of the article and by utilizing different names for the recovering substance user. The photo depicted their hands holding a syringe or pills without any additional identifiers that could signal other characteristics such as income-level (Doleac & Stein, 2013)—a depiction of substance users that is exceptionally common in articles about substance abuse. Figure 1 shows examples of two of these photos, varying by race, and all photos that we used are presented in Online Appendix C. We also varied the person’s name between one commonly attributed either to non-Hispanic whites or to African-Americans (Gaddis, 2017).^6^

We varied other identity attributes of the person depicted in the news story in simpler ways. We varied gender identity via the substance user’s name and the use of gendered pronouns in the news story. We varied the person’s residential location by describing the person as living in one of three alternate locations: a rural farm, a quiet suburb, or an urban downtown center.^7^ Finally, our experimental design also included two manipulations unrelated to group identity: how the substance user received treatment for their addiction (via public vs. private insurance) and the initial drug with which the person began using opioids (heroin, legally prescribed OxyContin, or illegally obtained OxyContin). We use the latter of these manipulations to assess one potential mechanism behind our main effects of identity.

We use three primary outcome variables. First, to measure support for opioid *treatment* policy, we asked each respondent their desired degree of change to federal funding for opioid treatment programs as follows: “If you were making up the budget for the federal government this year, would you increase, decrease, or keep spending the same for treatment for those addicted to opioids?” Second, to measure support for a *punitive* response to the opioid crisis, we asked each respondent their desired degree of change to federal funding for law enforcement activity as follows: “If you were making up the budget for the federal government this year, would you increase, decrease, or keep spending the same for law enforcement to arrest and prosecute those addicted to opioids?” Response options for both questions were “increase a lot,” “increase a little,” “keep the same,” “decrease a little,” and “decrease a lot.” Finally, we ask about individual blame and deservingness, a critical theoretical pathway through which racial identities have been shown to influence public opinion. We asked respondents: “Would you agree or disagree that individuals addicted to opioids are to blame for their own addiction?” The five response options ranged from “strongly agree” to “strongly disagree.”

We also measured several different characteristics of each survey respondent. We asked respondents for their demographic information and self-reported ZIP codes to code each respondent’s race, gender, partisan identity, and residential location.^8^ We also asked respondents whether they personally know anyone who has dealt with opioid addiction, or if they themselves have.

We fielded this survey on a nationally-representative probability sample of 3,112 adult respondents recruited via NORC’s AmeriSpeak Panel in June 2019.^9^ Specifically, the sample was selected from the AmeriSpeak panel by sampling within strata of age, race/ethnicity, education, and gender. In addition, the sampling strategy makes use of expected differential response rates in order to produce enhanced representation of “hard-to-reach rural households” (Dennis, 2016), allowing us to make refined estimates of respondent subgroup opinions among populations of special interest for this project. We present additional sampling details, as well as full descriptive statistics for our sample, in Online Appendix F.

## Results

We first analyze the treatment effects of each of our identity attribute manipulations across the entire survey sample. We tested for these effects by comparing the average support for increased treatment and law enforcement funding among the different treatment conditions. For each attribute that we varied, we examine the differential levels of policy support among respondents in each experimental condition. We code our main outcome variables, respondents’ desired increase or decrease in spending, as a continuous interval of support that takes a value of 1 if respondents strongly agreed to increase funding and a value of 0 if they strongly disagreed. For the mediation analysis, we similarly recode our outcome of individual blame with a value of 1 if respondents strongly agreed that those struggling with addiction are to blame for their own addiction and a value of 0 if they strongly disagreed.^10^

**Fig. 2:**
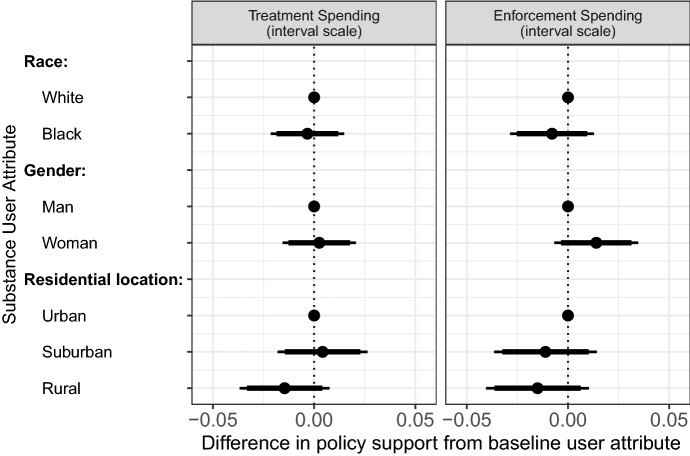
Treatment effects and confidence intervals among all respondents on unit scale interval outcome. Points are regression coefficients and indicate the difference in levels of policy support between respondents in the baseline level condition (no confidence interval) compared to respondents in conditions with all other attribute levels. Lines indicate 95%-confidence intervals (thin lines) and 90%-confidence intervals (thick lines)

Figure 2 plots our treatment effects of each attribute level among the full sample of respondents on our measure of support for treatment funding and law enforcement funding, with effects in the positive direction indicating greater support. For each attribute, we use one level as the reference category and show treatment effects of other attribute levels relative to that baseline.

Among our full sample of respondents, none of the three identity attributes of the substance user affected support for either treatment or law enforcement funding. Contrary to *H1*, respondents who read the news story about a substance user who was Black were no less likely to support increasing funding for treatment than those who read about a white substance user. Respondents who read the news story about a female substance user reported levels of support that were nearly identical to support among those who read about a male substance user. The residential identity of the substance user also had relatively small treatment effects. None of these differences were statistically significant among our full sample of survey respondents. The null effects of identity on support for treatment funding were nearly identical for law enforcement funding.^11^

### Moderation from Respondents’ Identities

However, our main theoretical expectation and corresponding pre-registered hypotheses held that the influences of these group identities on opinion would hinge on the respondents’ own identities. To assess this type of treatment effect heterogeneity, we next present the analyses of our treatment effects for race, gender, and residential location among subgroups of respondents. This allows us to assess descriptive moderation of treatment effects when a substance user’s identity matches that of the respondent.^12^ For each attribute of the substance user depicted in the news story in our experiment, we compare the treatment effect among the group of respondents whose own identity matches one attribute level to the effect among the group of respondents whose identity does not match that attribute level.

**Fig. 3:**
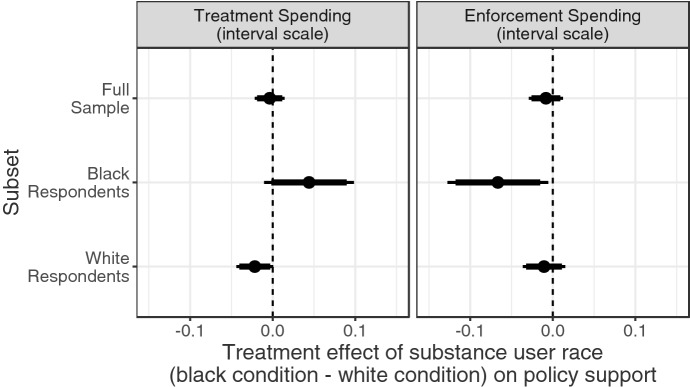
Treatment effects and confidence intervals by respondent race on unit scale interval outcome. Points indicate the difference in policy support between respondents who saw a Black individual profiled and a white individual profiled, with 95%-confidence intervals (thin lines) and 90%-confidence intervals (thick lines)

We first assess the degree to which respondents’ racial identity moderates the treatment effect of the substance user’s race. As described above, we observed an overall null treatment effect of the race of the substance user depicted in the news story on both policy outcomes, which we plot at the top of the two panels in Fig. 3. However, this treatment effect operates heterogeneously, as evidenced by the effects among respondents’ racial groups, which we plot in the middle and on the bottom of Fig. 3 for Black and white respondents, respectively.

For our treatment funding policy outcome, plotted in the left panel of Fig. 3, among Black respondents, those in the ‘Black’ treatment condition were 4 percentage points more likely to support treatment funding than those respondents in the ‘white’ treatment condition. In contrast, among white respondents, those in the condition depicting a Black substance user were 2 percentage points less supportive of a funding increase than those in the condition depicting a white substance user. The interaction between the experimental race manipulation and respondents’ race of 7 percentage points is statistically significant ($p = 0.022$).^13^ These effects replicate findings of shared racial identity from a pilot study we fielded on Amazon.com’s Mechanical Turk platform (see Online Appendix E). The larger magnitude of the effect of race among Black respondents on this outcome is particularly interesting, as it challenges existing theories that mainly concern the ingroup favoritism of *white* Americans—and their potential racial animus—when it comes to social policy opinions. Instead, these results support *H2a*, suggesting race-based ingroup favoritism for both white and Black respondents.

For our law enforcement spending policy outcome, plotted in the right panel of Fig. 3, we observed effects that mirror those of our treatment spending outcome. Black respondents in the ‘Black’ treatment condition were statistically significantly less likely to support increased enforcement spending than Black respondents in the ‘white’ treatment condition by 7 percentage points. We observed a small and statistically insignificant effect among white respondents. The interaction between the race manipulation and respondents’ race of 6 percentage points is not statistically significant at the 95% level ($p = 0.091$), though it suggests that respondent race may moderate the effect of racial identity on support for punitive policy as well as on treatment policy.

While these results present mixed evidence for *H3a*, they reflect those of Hurwitz and Peffley (2005a), who exposed respondents to descriptions of racial profiling and police brutality. The researchers observed that Black respondents who perceived the criminal justice system as chronically unfair exhibited ingroup favoritism in their judgements of the encounters, whereas white respondents were not sensitive to the race of the individual targeted. Hurwitz and Peffley (2005a) attributed this differential to the white respondents’ perception of ‘color-blind’ fairness in the criminal justice system. In contrast to Black Americans, many white Americans have not experienced similarly high levels of incarceration and its community-wide consequences (Western, 2006). Given this lack of exposure to the criminal justice system and its racial biases, white Americans’ current attitudes towards law enforcement in response to opioid use may not be as polarized by race, lead to the null effect among that subgroup.

**Fig. 4:**
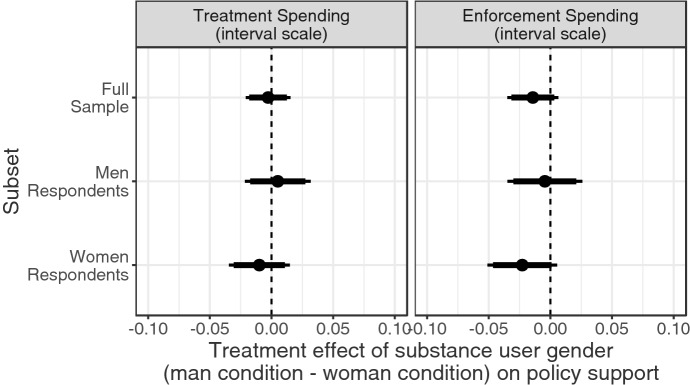
Treatment effects and confidence intervals by respondent gender on unit scale interval outcome. Points indicate the difference in levels of policy support between respondents who saw a male substance user profiled and those who saw a female substance user profiled, with 95%-confidence intervals (thin lines) and 90%-confidence intervals (thick lines)

We next assess the role of respondents’ gender identity in moderating the treatment effect of gender, which we show in Fig. 4. As described in the previous section, among the full sample of respondents, we observed a null treatment effect of the gender of the substance user depicted in the news story on both policy outcomes, as plotted at the top of Fig. 4. Contrary to *H2b*, neither men nor women showed ingroup preferences on our treatment spending outcome. Likewise, a shared gender identity has a similar null effect on our enforcement spending outcome, suggesting that respondents’ gender does not moderate the effect of gender identity on support for punitive spending (*H3b*).

**Fig. 5:**
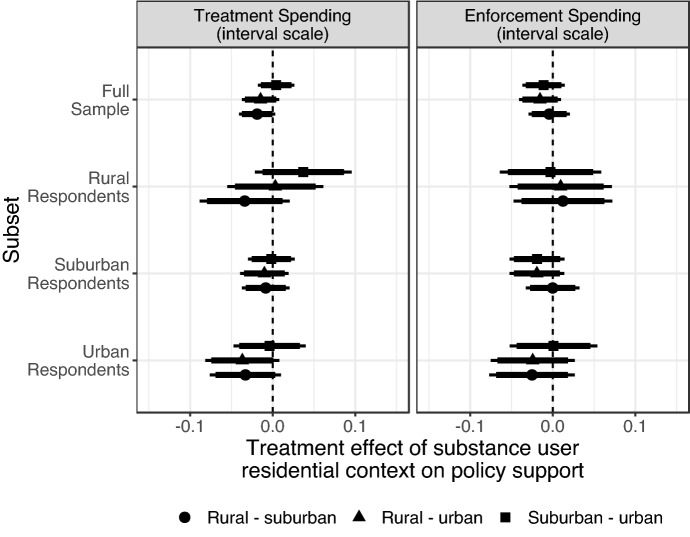
Treatment effects and confidence intervals by respondent geographic context on unit scale interval outcome. Points indicate the difference in policy support between respondents who saw a rural individual profiled vs. a suburban individual profiled (circles), rural vs. urban individual (triangles), or suburban vs. urban individual (squares), with 95%-confidence intervals (thin lines) and 90%-confidence intervals (thick lines)

Finally, we conduct similar analyses of the treatment effect of residential context among respondent subgroups of residential context. We plot the effects of the residential context of the individual portrayed in the article for our treatment spending outcome in the left panel of Fig. 5 and for our law enforcement spending outcome in the right panel, using separate shapes for each of the three comparisons between experimental conditions. We show these effects for our full respondent sample on the top, among respondents in rural locations (second from the top), suburban locations (third), and urban locations (on the bottom). For our first policy outcome, among the full sample of respondents, reading about a rural versus an urban substance user had a null effect, as described earlier. Within each geographic subgroup, none of the effects were statistically significant, meaning respondents showed little ingroup favoritism for people from their own residential context (*H2c*). For our second outcome, support for law enforcement spending policy, we see a similar lack of subgroup effects (*H3c*). Together, these results show that shared residential identity on its own is unlikely to shape opinions on opioid-related policies.

**Fig. 6:**
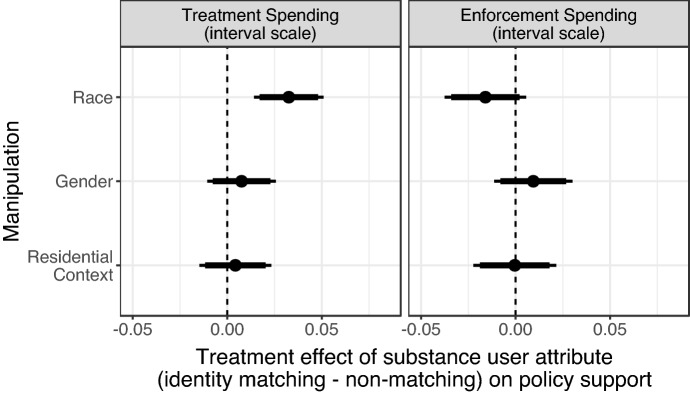
Treatment effects and confidence intervals for match between respondent characteristic and substance user attributes on unit scale interval outcome. Points indicate the difference in each policy outcome between respondents who matched the individual profiled and those who didn’t match them for each of the three identity attributes, with 95%-confidence intervals (thin lines) and 90%-confidence intervals (thick lines)

To assess the effects of identity across multiple respondent subgroups simultaneously, we also examined whether the match between one aspect of a respondent’s identity and the identity of the person profiled in the article had an effect on their policy support—essentially, aggregating across respondent subgroups to assess ingroup or outgroup favoritism for each demographic characteristic. These effects are plotted in Fig. 6. Each of the points in this figure is based on differences in opinions between respondents whose identity matches that of the individual in the media story along that one identity attribute, and respondents whose identity does *not* match that attribute. While matching the identity of the substance user depicted in the news story appears to have a uniformly positive effect on support for treatment spending, this ingroup bias is only statistically distinguishable from zero for racial identity. We see more muted racial ingroup favoritism effects for respondents’ support of the punitive law enforcement spending policy, which would be reflected by negative coefficients for the punitive outcome. In short, shared identity on attributes outside of race does not affect support for either opioid-related policy.

### Mechanisms

We next assess one potential mechanism behind our results: perceptions of personal blame. To do so, we use the question on our survey asking respondents the degree to which they believed substance users like the one depicted in the story are to blame for their own drug addiction. Overall, 52 percent of respondents somewhat or strongly agreed with the statement that those addicted to opioids are to blame for their addiction. As hypothesized, agreement with this attribution of blame was highly predictive of policy opinions (*H4*). Those who agreed with the statement were 17 percentage points less likely to support treatment spending policy and 16 percentage points more likely to support law enforcement spending policy than those who did not think individuals were to blame.

To more formally test whether this perception of blame acts as a crucial causal mechanism behind the effect of group identity on policy opinions, we conducted exploratory causal mediation analyses using blame as a continuous variable (Imai et al., 2010, 2011). These analyses rest on an assumption of sequential ignorability that is potentially untenable in this and many other situations, and a different experimental design could yield a superior method of assessing blame’s mediating role by randomizing the mediator in a parallel design (Imai et al., 2011) or setting values of the mediator (Acharya et al., 2018). Yet these analyses do provide an initial exploration of the plausibility of this mechanism. Contrary to *H5*, these analyses (which we present in Online Appendix L) suggest that none of the treatment effects associated with group identity were mediated by blame. This is despite the fact that the measure is predictive of policy opinions, and that we did observe variation on the measure itself between respondents.

The absence of evidence supporting blame as a mediator is surprising given the theoretic importance of perceived deservingness in support for social welfare policy. To further interrogate this, we assess the effects of identity within levels of one of our other experimental manipulations that was *not* based on substance users’ identity. We conduct several exploratory analyses that were not in our pre-analysis plan but are helpful in developing a more comprehensive understanding of how identity and blame intersect. Specifically, we analyze the effects of our manipulation that varied the initial drug which the person profiled in our story began using before becoming addicted to opioids.

**Fig. 7:**
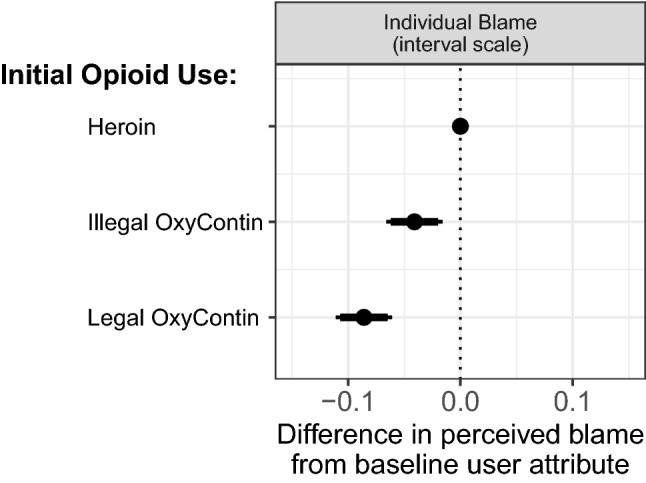
Treatment effects and confidence intervals among all respondents on unit scale interval outcome. Points are regression coefficients and indicate the difference in levels of perceived blame between respondents in the baseline condition (no confidence interval) of initially using heroin compared to respondents in conditions where the substance user was described with different initial drug use of either illegally-obtained OxyContin or legally prescribed OxyContin. Lines indicate 95%-confidence intervals (thin lines) and 90%-confidence intervals (thick lines)

First, we assess the common wisdom that many opioid users are considered less blameworthy because they began taking opioids legally, via a prescription. Figure 7 plots the effect of the initial drug manipulation on respondents’ perceptions of the blame that the substance user had for their situation. Our results support the folk wisdom, and show that respondents perceived people who were described as initially using OxyContin that was prescribed by a physician as less blameworthy compared to people who were described as using heroin. Substance users who were described as having first used either legal or illegal OxyContin were perceived by respondents to be 5 to 10 percentage points less blameworthy than those substance users described as first using heroin.

**Fig. 8:**
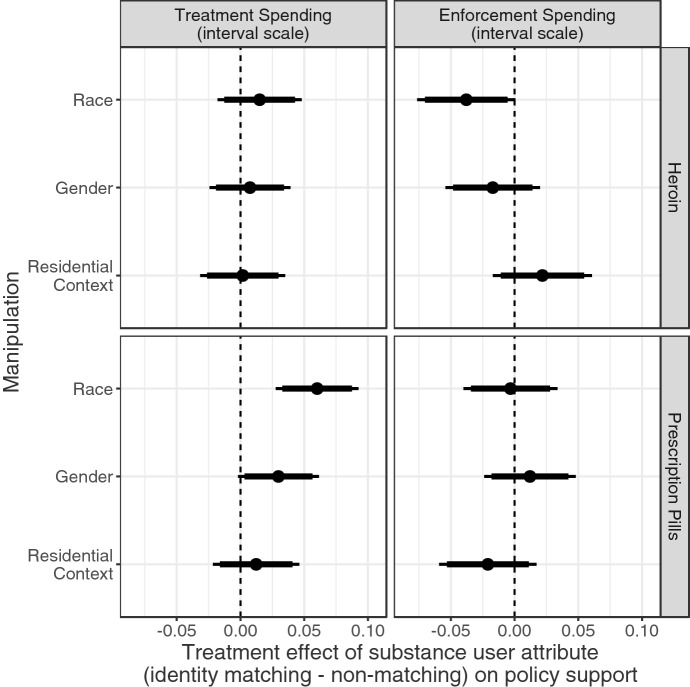
Treatment effects and confidence intervals for match between respondent characteristic and substance user attributes on unit scale interval outcome. Points indicate the difference in each policy outcome between respondents who matched the individual profiled and those who didn’t match them for each of the three identity attributes, with 95%-confidence intervals (thin lines) and 90%-confidence intervals (thick lines)

Given that respondents ascribe a higher level of individual blame to substance users who first used heroin, we next explored whether this manipulation might condition the effects of identity that we previously described. We examine this in Fig. 8, which replicates Fig. 6 and shows the effect of a shared identity on our policy outcomes but separately for respondents who read about a substance user who initially used heroin (top row) vs. legally-prescribed OxyContin (bottom row).^14^ For this first group, substance users who began their addiction by using heroin, perceptions of individual blame are much higher. In this condition, it appears that shared identity does not affect support for treatment funding. We see suggestive evidence that shared racial identity may decrease support for funding law enforcement ($p<0.1$).

For the second group of substance users, who began using opioids when prescribed OxyContin—and therefore were viewed less blameworthy—we see different effects. Sharing an identity with the substance user increased support for treatment funding by 6 percentage points ($p < 0.01$) while a shared gender identity had a smaller but still positive effect. These effects of shared identity only affected support for treatment funding and not punitive enforcement funding opinions.

These interaction results, in tandem with the ACME results described previously, suggest that identity may not have an effect on policy opinions *through* (i.e., mediated by) individual blame. However, blame may still *moderate* the effect of a shared identity. Few or none of the substance users described as initially using heroin, who were perceived to have high levels of blame, may be seen as worthy of treatment. Thus, a shared identity may not meaningfully affect support for funding sympathetic treatment programs. At the same time, some of the users associated with heroin may be viewed as more deserving of punishment than others. Given the primacy of race in conditioning criminal justice attitudes, we should not be surprised that a shared racial identity decreases support for funding law enforcement. In contrast, few or none of the substance users described as initially using prescription OxyContin, who were viewed as less blameworthy, may be seen as deserving punishment at all, limiting the influence of shared identity on respondents’ support for punitive policy responses. However, respondents may have room in their opinions to be selective in their support of treatment funding. Thus, these results reflect the established importance of identity in allocating group-based benefits, but suggest that there may be a moderating—if not mediating—role for perceptions of blame.

## Conclusion

In this paper, we have provided comprehensive evidence of the direct effect of media depictions of substance users on public opinion about opioid policy solutions. Our findings suggest that, much as with other social policies, racial group prejudice plays a large role in the way that people form opinions about both treatment- and punishment-focused opioid policies. Even given the cross-cutting nature of the opioid epidemic, where theory would lead us to say other group identities should matter in the formation of public opinion, race matters most.

Our results have several important limitations. Not all people will, in the real world, read depictions of individuals suffering from substance use disorder in the media. Thus the types of effects we observe may have different effects when consumed in a real-world setting. Information, and in particular information about the opioid crisis, may have heterogeneous effects based on the preferences individuals hold for consuming said information (Testa et al., 2020). The effects of racial identity on policy opinions that we do observe may even be an underestimate of the potential effects of identity given our relatively mild rates of passage on our manipulations checks (Kane & Barabas, 2019). Given this, the magnitude of our treatment effects—even if taken as a “treatment-on-treated” effect on a captive group of respondents—indicate the large potential for broader public opinion changes due to exposure to a single media story about this issue.

Additionally, though personal exposure to individuals with substance use may be one way that people learn about the effects of the opioid crisis (e.g., Kaufman & Hersh, 2020), news media are another way that people learn about real-world conditions around themselves (e.g. Neuner et al., 2019). Indeed, personal news consumption is positively associated with the perceived severity of the opioid epidemic in one’s community (Gollust & Haselswerdt, 2021). For the large sector of the population that is not directly affected by the crisis, the information conveyed in these media stories may be the most important consideration in the formation of their policy opinions.^15^

Our evidence that mass opinion is subject to biases based on racial identity not only affirms the centrality of group identity in policy opinions, but also highlights a potential fault in representation. Policymakers are frequently biased in whose preferences they are responsive to, favoring whiter and wealthier constituents in their policy decisions (e.g. Butler & Broockman, 2011; Gilens, 2012). Likewise, lawmakers are constrained in their capacity to handle urgent crises like the opioid crisis that demand regulatory oversight of local implementing authorities (e.g. Cook & Fortunato, 2022; Fortunato & Parinandi, 2022). Especially under such capacity constraints, legislators may only act to pass and implement policy when they see a crisis as more urgent—a perception that may be shaped by biases favoring certain groups, as we show. This might lead to lawmakers ignoring the need for policy action when it affects other groups to which they are less sympathetic. Policymakers should therefore be attentive to the effect of media narratives on public opinion when creating policy if they wish to adhere to principles of democratic representation.

## Supplementary Information

Below is the link to the electronic supplementary material.

Supplementary file1 (PDF 15049 kb)

## Footnotes

1 The pre-analysis plan for this study was registered prior to data collection at EGAP (# 20190515AD) and is included in Online Appendix N. The study was conducted in compliance with relevant laws and the research design was approved by our institution’s IRB, and informed consent was obtained from all individual participants included in the study. The data and replication code for our study can be found at: https://doi.org/10.7910/DVN/QMVU0Z.

2 Though we do not asses it in this paper, the racial identity of people depicted in media stories about the opioid crisis may also shape perceptions of which groups are “losing” in the political and public health arenas (Gollust & Miller, 2020), which might subsequently shape policy opinions.

3 Full wording of the article and all survey questions is in Online Appendix A.

4 A summary of all experimentally varied attributes and levels of each attribute are described in Online Appendix B.

5 This decision may limit the generalizability of our findings to Latinos and Asian-Americans, whose group identities are prone to be based on national-origin and immigration status as well as ethnicity/race (Masuoka & Junn, 2013).

6 To account for socio-economic factors within race, we selected two names for each race-gender pairing, with a name from both the lowest and highest education quartile within each race.

7 We include a series of factual manipulation checks for each of these manipulations, which indicate our manipulations were overwhelmingly effective at shifting the perceived identity characteristics (Online Appendix D).

8 We code residential location as either ‘urban,’ ‘suburban,’ or ‘rural’ based on the population density classifications described by Kolko (2015).

9 We based our sample size on estimates of statistical power from a pilot survey we fielded in January 2019 (Online Appendix E).

10 Results are nearly identical when using binary coding of the outcome scales rather than the continuous interval coding of the dependent variables (Online Appendix G).

11 Results for our additional manipulations (insurance coverage and pathway to addiction) are reported in Online Appendix H. While not pre-registered, we also assess the potential for interactions among our treatments (Online Appendix I) as well as conditional effects based on the respondent’s partisanship (Online Appendix J). Finally, we report the estimated false discovery rate for each hypothesis test given multiple hypothesis testing (Online Appendix K).

12 All conditional treatment effects are simply subgroup differences in the causal effects of our attribute manipulations, and do not necessarily represent *causal* differences of these respondent characteristics.

13 This test for the moderation of treatment effects follows our pre-registered method for testing for this difference using an interaction between the race condition and respondent race in OLS.

14 For the sake of brevity, we only conduct these exploratory analyses for the two conditions of initial drug use for which we see more extreme differences on perceived blame, as shown in Fig. 7.

15 Analyses of the effects of group identity on policy opinions within subgroups of personal exposure to the crisis, however, suggest that even those who know someone struggling with addiction may still be influenced by these factors (see Online Appendix M).
