# Supplementary material for: How the Identity of Substance Users Shapes Public Opinion on Opioid Policy
Source: Polit Behav. 2022 Dec 20:1–21. Online ahead of print. doi: 10.1007/s11109-022-09845-8 (PMC9765388; doi:10.1007/s11109-022-09845-8)
Supplement: Supplementary file 1 — Supplementary file1 (PDF 15049 kb) [file 11109_2022_9845_MOESM1_ESM.pdf]

Supplementary Appendix for "How the Identity of Substance Users Shapes Public Opinion on Opioid Policy"

Justin de Benedictis-Kessner*   Michael Hankinson†

December 8, 2022

*Assistant Professor, John F. Kennedy School of Government, Harvard University. 79 John F. Kennedy St., Cambridge, MA 02138. jdbk@hks.harvard.edu

†Assistant Professor, Department of Political Science, George Washington University. 2115 G Street, N.W., Washington, D.C. 20052. hankinson@gwu.edu

Contents

A Survey Instrument A-1

B Experimental Manipulations A-4

C Photos from Experiment A-5

D Results from Manipulation Checks A-7

E Power Analyses from Pilot Study A-9

F Descriptive Characteristics of Survey Respondents A-12

G Results Using Alternative Coding of DV A-13

H Additional Treatments A-18

I Interaction Effects of Randomized Treatments A-19

J Heterogeneity in the Effects by Respondent Partisanship A-27

K Multiple Hypothesis Testing Corrections A-29

L Mediation Analyses A-36

M Heterogeneity in the Effects of Group Identity by Personal Exposure A-38

N Pre-Analysis Plan A-41

A Survey Instrument

Experimental Vignette

The following replicates the introduction and news story all subjects read, with text varying according to the experimental condition. All gendered pronouns varied based on gender randomization, but example uses female pro-nouns for clarity.

We are interested in learning what people can remember from what they read in news articles. We would now like you to read a news article, and then answer some questions about it. You will find the article on the next page. Please read it carefully before answering the following questions.

There will be a brief pause on the next screen so you can read the story. At the end of the pause, an arrow will appear at the bottom of the screen.

Once the arrow appears, you may move on to the next screen of the survey by clicking on the arrow.

PHOTO OF HAND HOLDING DRUG PARAPHERNALIA
(See Section C for photos.)

NAME, holding the drugs that started her addiction.

NAME is a resident of CONTEXT and a recovering opioid addict who has witnessed the disturbing, dark side of addiction.

Growing up, NAME had what appeared to be a bright future ahead of her. But after high school, NAME got her first taste of the drug that would come to rule over her life. PATHWAY.

“It was instant love,” she said. “That was the first time I got that opioid feeling, and I really liked the way it felt.”

Her life started to go downhill, quickly. While most of the people she grew up with were graduating from college or getting their first big job, she was doing whatever she had to do to get high. After her first introduction to opioids, she started buying the drugs illegally from people she knew were selling in her town. To pay for these drugs, she did things she now says she regrets given the toll they took on her life. Over the past four years, NAME overdosed twice. Both times she was found by friends or family members and revived by paramedics or in the hospital.

“About a year ago, I just finally had enough and hit a point where either I stopped or I was going to die and not come back this time,” she said. NAME is now in outpatient rehab. She is thankful for her INSURANCE. Otherwise, she would not be able to afford treatment and could not begin her journey to recovery.

”I just hope people will hear my story and realize there is a way out. You just have to want it. The disease may not have been my responsibility, but the recovery is 100 percent my responsibility,” she said.

Outcome Variables

We randomize the order of questions 1 and 2 (“treatment” and “law enforcement to arrest and prosecute”).

Now, we would like to know your opinion about opioid treatment programs.

1. If you were making up the budget for the federal government this year, would you increase, decrease, or keep spending the same for treatment for those addicted to opioids?
    - Increase a lot
    - Increase a little
    - Keep the same
    - Decrease a little
    - Decrease a lot

2. If you were making up the budget for the federal government this year, would you increase, decrease, or keep spending the same for law enforcement to arrest and prosecute those addicted to opioids?
    - Increase a lot
    - Increase a little
    - Keep the same
    - Decrease a little
    - Decrease a lot

3. Would you agree or disagree that individuals addicted to opioids are to blame for their own addiction?
    - Strongly agree
    - Somewhat agree
    - Neither agree nor disagree
    - Somewhat disagree
    - Strongly disagree

Manipulation Checks

Due to survey length constraints, two of the five manipulation checks were randomly selected for each respondent to answer.

Next, we are going to ask you a few questions about the individual profiled in the news article you read about opioid addiction.

- What was the race of the individual profiled in the news article?
    - Asian
    - Black
    - Hispanic
    - White
- What was the gender of the individual profiled in the news article?
    - Male
    - Female

- In which type of community did the individual profiled live?
  - A rural farm
  - A quiet suburb
  - An urban downtown center
- How did the individual profiled become addicted to opioids?
  - Injured his/her knee and needed surgery. His/her doctor prescribed him/her OxyContin pills for the pain during his/her recovery.
  - His/her friend illegally gave him/her OxyContin pain pills at a party.
  - His/her friend gave him/her heroin at a party.
- What type of insurance did the individual profiled have?
  - Insurance purchased from a private provider
  - Insurance purchased through the Affordable Care Act/Obamacare marketplace
  - Insurance coverage from their state's Medicaid expansion, funded by the Affordable Care Act/Obamacare

Demographic Variables

We collect demographic data on race, gender, ideology, partisanship, homeownership, age, and ZIP code from NORC panel variables. We also included a question on respondents' personal exposure to individuals struggling with addiction:

- Do you personally know anyone who has ever been addicted to opioids, including prescription painkillers or heroin? Please select all that apply.
  - Yes, me
  - Yes, a family member
  - Yes, a close friend
  - Yes, an acquaintance
  - No, I do not know anyone who has ever been addicted to opioids

B Experimental Manipulations

We varied the following attributes of the individual profiled in the news story, with full randomization that allowed each attribute to take one value with no restrictions based on other attribute values.

1. **Race** - name and use of dark-skinned or light-skinned hand in photo. We use names from the lowest education quartile and highest education quartile within race (e.g., Gaddis, 2017) to mitigate any socio-economic effects outside of race.
    - Black woman - Lakisha (lowest quartile), Janae (highest quartile)
    - White woman - Angie, Katelyn
    - Black man - DaShawn, Darius
    - White man - Ronny, Jake
2. **Gender** - name and use of he/she pronouns
3. **Residential location**
    - a rural farm
    - a quiet suburb
    - an urban downtown center
4. **Pathway to addiction** - story of person varied according to below options, along with drug paraphernalia depicted in image (i.e. when story described a person who began their opioid use with OxyContin pills, the image showed a hand holding pills, whereas when the story described a person who began their opioid use with heroin, the image showed a needle).
    - Injured his/her knee and needed surgery. His/her doctor prescribed him/her OxyContin pills for the pain during his/her recovery.
    - His/her friend illegally gave him/her OxyContin pain pills at a party.
    - His/her friend gave him/her heroin at a party.
5. **Insurance coverage**
    - insurance purchased from a private provider
    - insurance purchased through the Affordable Care Act/Obamacare marketplace
    - insurance coverage from the state's Medicaid expansion, funded by the Affordable Care Act/Obamacare

C Photos from Experiment

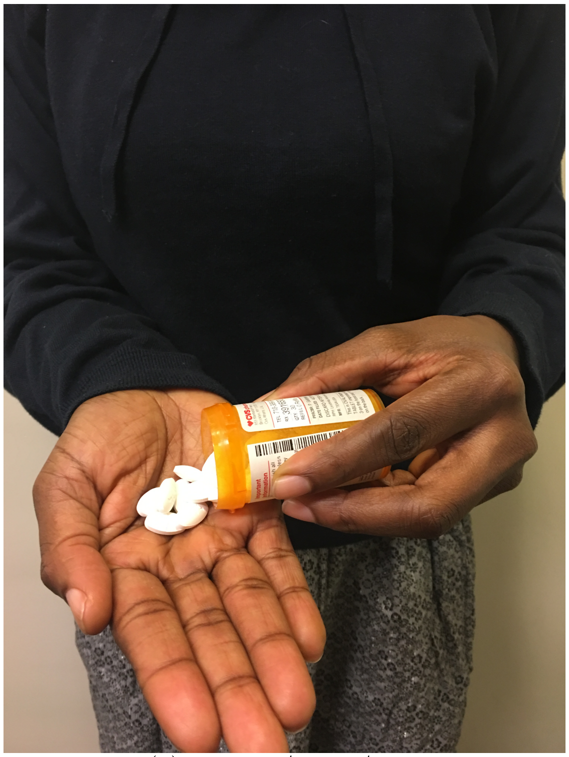

(a) Woman/Black/Pills

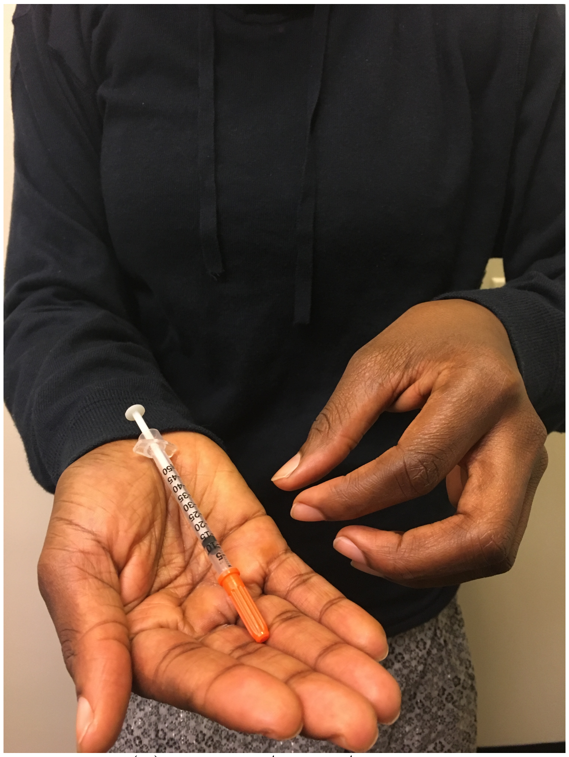

(b) Woman/Black/Needle

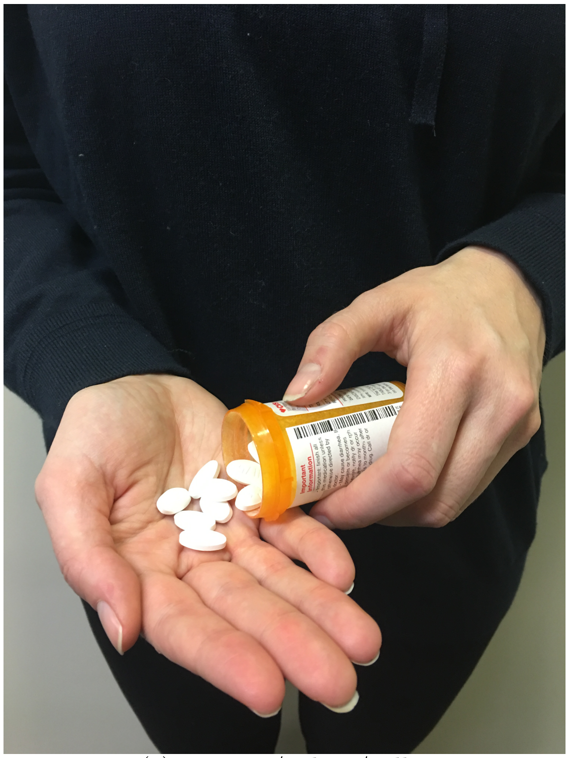

(c) Woman/White/Pills

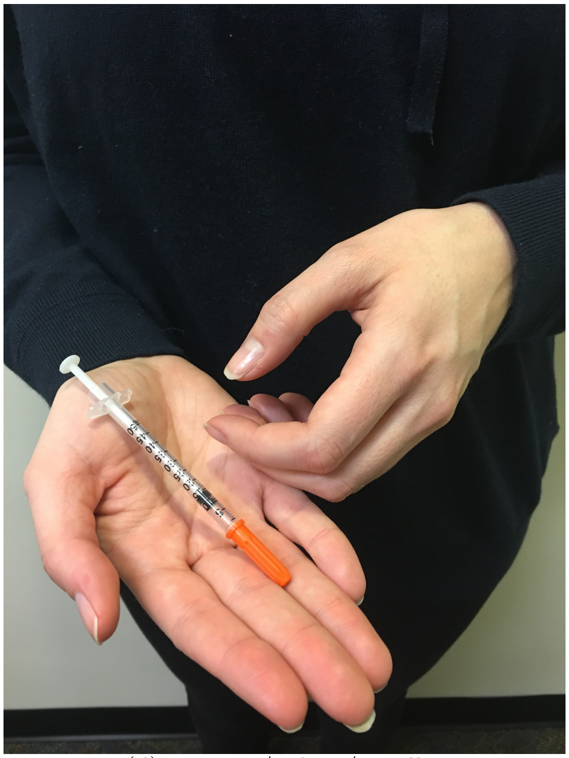

(d) Woman/White/Needle

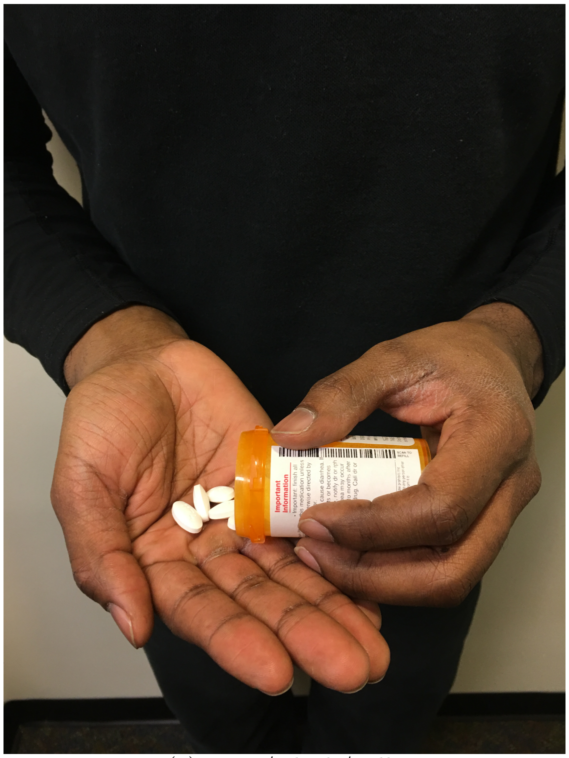

(e) Man/Black/Pills

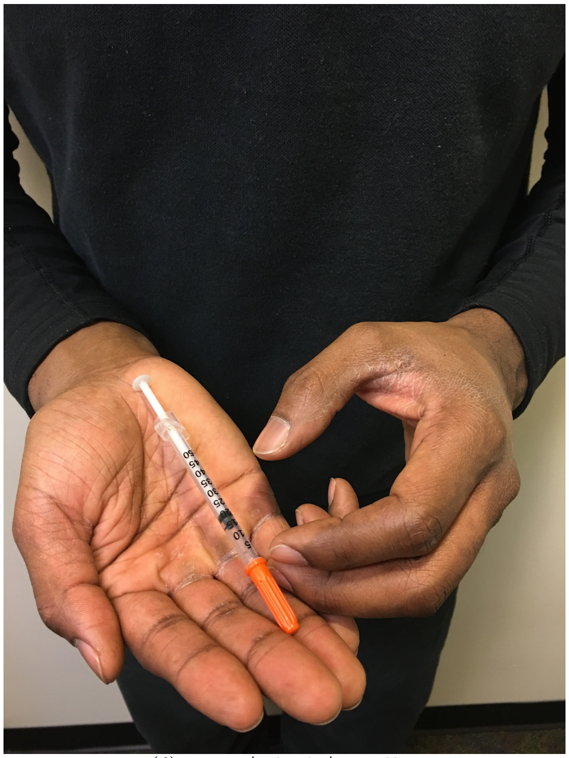

(f) Man/Black/Needle

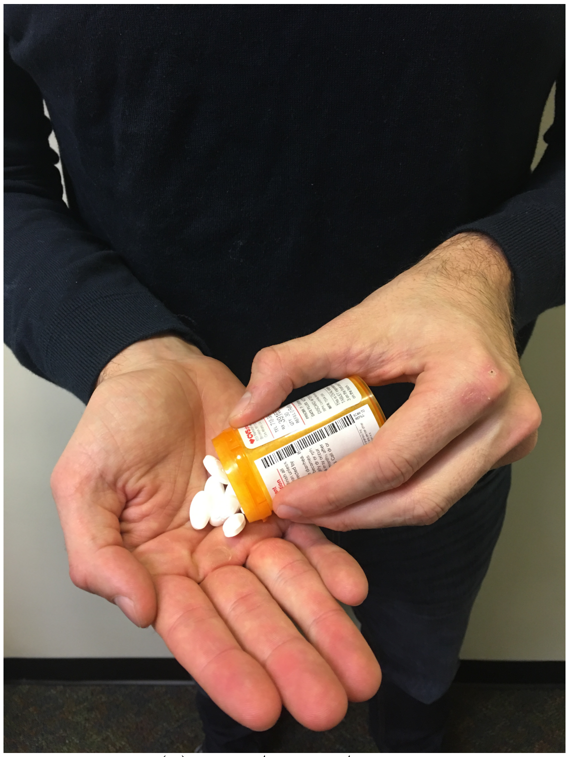

(g) Man/White/Pills

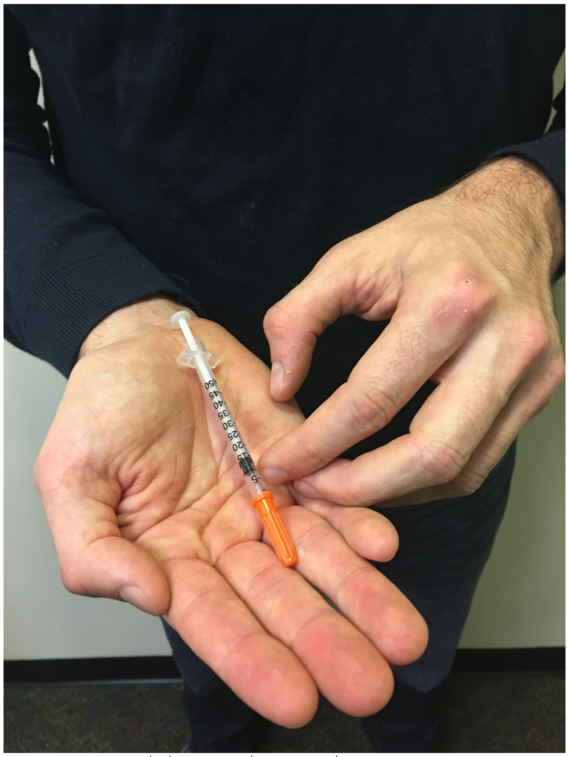

(h) Man/White/Needle

D Results from Manipulation Checks

In Figure D-1 we present the treatment effect of varying attributes on the proportion of respondents answering the manipulation check question in line with that treatment. Each point plots this treatment effects for a different attribute along with its 95% confidence interval. Each attribute that we varied greatly increased the proportion of respondents answering that option on the manipulation check question, and all treatment effects were statistically significant. For instance, respondents in the 'black' treatment group were 58 percentage points more likely to answer that the person profiled in the article they read was black than respondents in the 'white' treatment group ($p < 0.01$), as plotted with the left-most point. All other treatments had similarly large and statistically significant effects on our manipulation check questions, indicating that our experimental manipulations were conveying the information we hoped to convey.

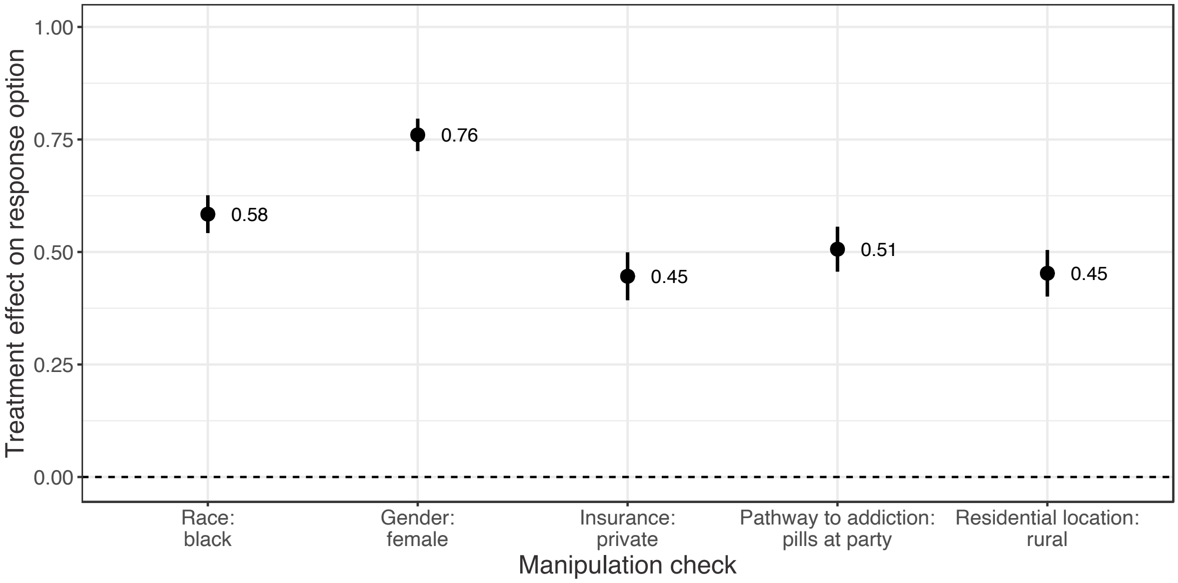

Figure D-1: Manipulation checks by experimental attribute. Points indicate the percent of respondents correctly recalling the attributes of the individual profiled.

We note that these strong treatment effects on our manipulation check recall questions do, however, accompany relatively low overall "passage rates" on some of these questions, potentially due to strong underlying assumptions that respondents have about substance users and the traits that we experimentally manipulate. Figure D-2 shows the proportion of respondents recalling each relevant attribute for our main manipulations. As Kane and Barabas (2019) note, however, we should interpret significant differences in passage rates between conditions (i.e. the ATEs presented above) alongside these modest passage rates for manipulation checks as indicating that our treatment is "strong enough to exert an effect *despite* a sizable presence of inattentive respondents" (p. 238). In other words, the presence of treatment effects on our manipulation check outcomes but overall low levels of correct recall on these manipulation checks should suggest that our experimental treatment effects are an *underestimate* of what treatment effects on policy attitudes might be if there were more perfect compliance.

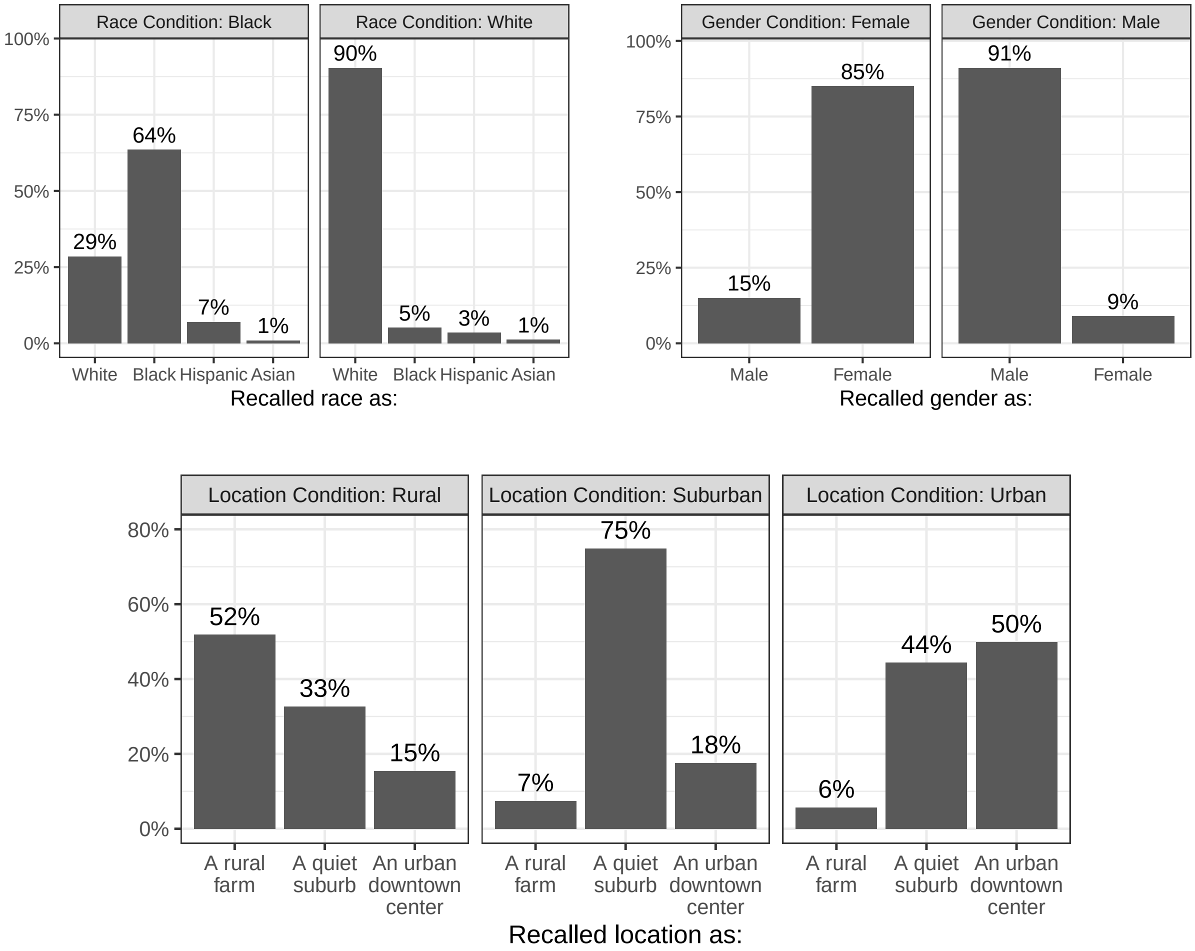

Figure D-2: Manipulation check responses by experimental condition.

E Power Analyses from Pilot Study

In January 2019, we fielded a nearly identical experiment on Amazon.com's Mechanical Turk (MTurk) platform as a pilot study ($n = 800$). The pilot treatment effects that we observed on our limited sample enabled us to conduct power analyses helping inform the necessary sample size for the study. As shown below, the effects of shared racial identity on opioid policy support that we report in the main text replicate our pilot findings from the smaller MTurk sample. In contrast, our study did not find similar effects of shared residential identity as found in the MTurk sample. Additionally, our pilot study did not include outcome variables on punitive policy or deservingness. Still, the pilot findings informed the sample size we believed necessary to replicate the effects using the NORC's AmeriSpeak sample ($n = 3,112$).

Reviewing the pilot study data, we first analyzed the treatment effects on our race manipulation. We tested for this effect by comparing the average support for treatment funding — which, as in the main text, we recoded as a continuous measure of support — among respondents who read the profile of a black person struggling with addiction to the support for spending among respondents who read the same profile of a white person. This difference in means represents the treatment effect of race.

We plot the treatment effect for the full sample of respondents on the left side of Figure E-3. Within this pilot sample, we observed an overall null treatment effect: respondents in the 'black' condition were 1 percentage points less likely to support funding than respondents in the 'white' condition. However, as we hypothesized, this treatment effect should operate heterogeneously, with respondent's identity moderating the overall effect. In this case, we expected that respondents' racial identity would do so and consequently tested for the heterogeneity of these effects by respondents' race.

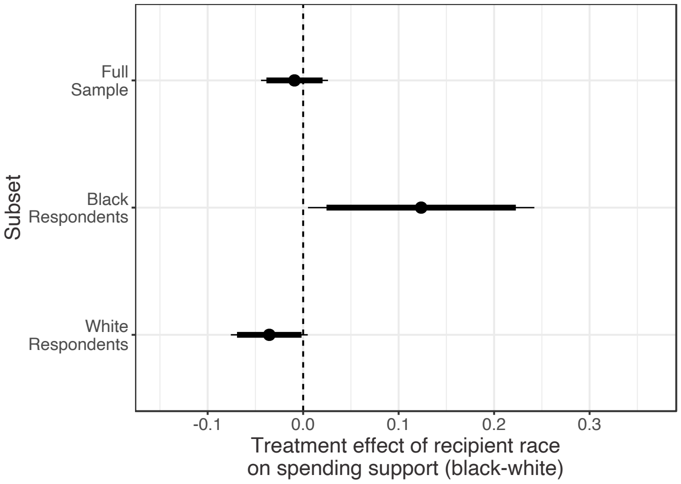

Figure E-3: Pilot study treatment effects and confidence intervals by respondent race. Points indicate the difference in levels of support between respondents who saw a black individual profiled and a white individual profiled, with 95%-confidence intervals (thin lines) and 90%-confidence intervals (thick lines).

Among black respondents, those in the 'black' treatment condition were 12 percentage

points more likely to support funding than those respondents in the 'white' treatment condition. We plot this treatment effect in the middle of Figure E-3, which is statistically significant ($p = 0.041$). Using the standard deviation of the outcome variable among black respondents, we calculate this as a standardized effect size of 0.42. Among white respondents, we observe a treatment effect of a 4 percentage point decrease in support for funding ($p = 0.084$), which we plot on the right side of Figure G-6. This is a standardized effect size of 0.14. Like our AmeriSpeak panel sample, the effects are nearly identical when operationalizing support for treatment spending as a binary variable.

Using these two standardized effect sizes, we conducted power analyses to test what the required sample size would be in each respondent racial group to detect effects of these sizes with an α of 0.05 and power of 0.8. This yielded a required treatment condition size of 90 among black respondents and 766 among white respondents, yielding a total required n of 1712.

Though this power analysis indicated a need for a relatively small sample size, we also wanted to test for the required sample size to detect some smaller treatment effects on other experimentally manipulated attributes of the person profiled in the article with a greater number of potential attribute values — for instance, the person's residential context — and therefore a greater number of experimental conditions. We conducted similar analyses of this treatment effect in our pilot, among our full sample (plotted on the left of Figure E-4) and among respondents in rural locations (in the middle of Figure E-4) and non-rural locations (right side of Figure E-4).[1] As with our tests of the effect of race, we operationalize the treatment effect of the residential context of the person profiled in the article as the difference in support for treatment funding among those respondents who viewed the profile of a rural individual (from 'a rural farm') and support among those respondents who viewed the profile of a non-rural individual (from 'a quiet suburb' or 'an urban downtown center').

On average, the geographic context of the individual profiled had a null effect on respondent support for treatment funding. However, among rural respondents, we find a 7 percentage point increase in funding support (p=0.188). We observe a small negative treatment effect among non-rural respondents, but it is statistically indistinguishable from zero with this sample size. While these effects are not statistically significant, they match the shared identity relationships we found for race.

We used these pilot treatment effect sizes to also conduct power analyses for this experimental treatment. In order to detect a treatment effect of residential context of the sizes observed in our pilot among rural respondents, we would have needed to have 255 respondents in each condition, while we would need 2218 non-rural respondents in each condition in order to detect negative effects of the size observed in our pilot. Though this yielded a clearly unrealistic required sample size of 4946, we believed that there were several reasons to still test for this treatment effect on a large nationally representative sample.

For one, estimates of heterogenous effects — a primary quantity of interest in this study — can suffer substantially from the idiosyncratic features of a Mechanical Turk sample (e.g., Berinsky, Huber, and Lenz, 2012; Levay, Freese, and Druckman, 2016). Geographic variation on MTurk is unreliable, and suggests that our opinion estimates among rural and non-rural

[1]We use respondents' ZIP codes to group them into rural and non-rural subgroups and test for context-based in-group preferences among these respondents reflective of Cramer's (2016) theory of 'rural consciousness.'

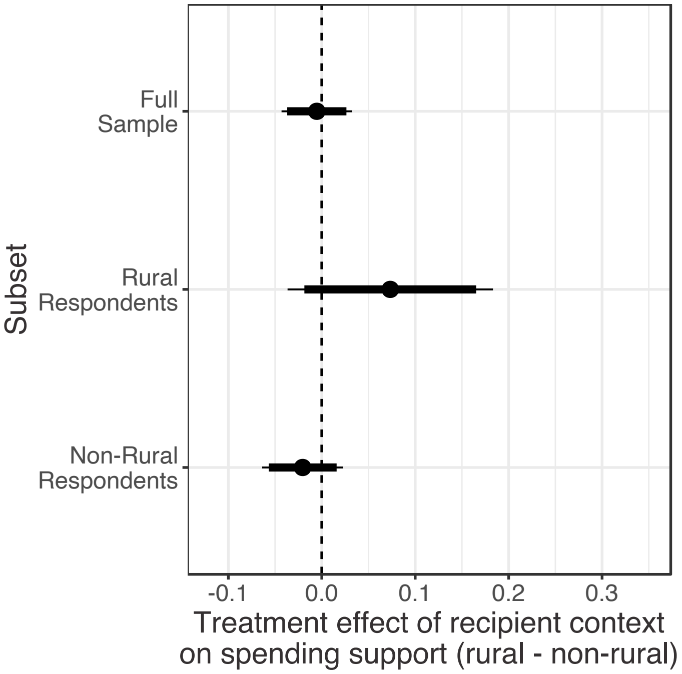

Figure E-4: Pilot study treatment effects and confidence intervals by respondent geographic context. Points indicate the difference in levels of support between respondents who saw a rural individual profiled and a non-rural individual profiled, with 95%-confidence intervals (thin lines) and 90%-confidence intervals (thick lines).

respondents may have been biased, leading to potential biases in our estimated treatment effects. A TESS study fielded via NORC's AmeriSpeak Panel, on the other hand, would make use of enhanced representation of "hard-to-reach rural households" (NORC, 2016). This would help us refine our estimates of opinion among rural and non-rural respondents, as well as the other respondent subgroups of particular interest for this study. Thus, we pursued a nationally representative sample of over 3,000 respondents via NORC, the results of which are reported in the main text of this paper.

F Descriptive Characteristics of Survey Respondents

As described in the main text of the manuscript, NORC drew a stratified probability sample from their AmeriSpeak Panel to invite to participate in our survey. The survey was in the field from June 16 to July 11, 2019, during which period NORC sent 5 reminder emails and one SMS reminder. Panelists were offered the cash equivalent of $1 to complete the survey via NORC's points incentive system. The median respondent took 3 minutes to complete the survey. The weighted AAPOR RR3 response rate was 30.3%.

Below, we present descriptive statistics of interest for our full sample of survey respondents.

Table F-1: Sample Descriptive Statistics

Statistic	Mean	St. Dev.	Median	Min	Max	N
Female	0.51	0.50	1	0	1	3,112
White	0.65	0.48	1	0	1	3,112
Black	0.12	0.33	0	0	1	3,112
Age	48.06	17.04	47	18	92	3,112
Homeowner	0.64	0.48	1	0	1	3,112
Urban location	0.25	0.43	0	0	1	3,112
Suburban location	0.58	0.49	1	0	1	3,112
Rural location	0.17	0.37	0	0	1	3,112
Personal exposure	0.59	0.49	1	0	1	3,112

G Results Using Alternative Coding of DV

In this section, we replicate the main results with an alternative coding of the dependent variables as binary measures of support that take a value of 1 if respondents supported increasing funding by a lot or a little and a value of 0 otherwise. These alternative results, presented below in Figure G-5, are largely similar to those presented in the text of the paper in Figure **??**.

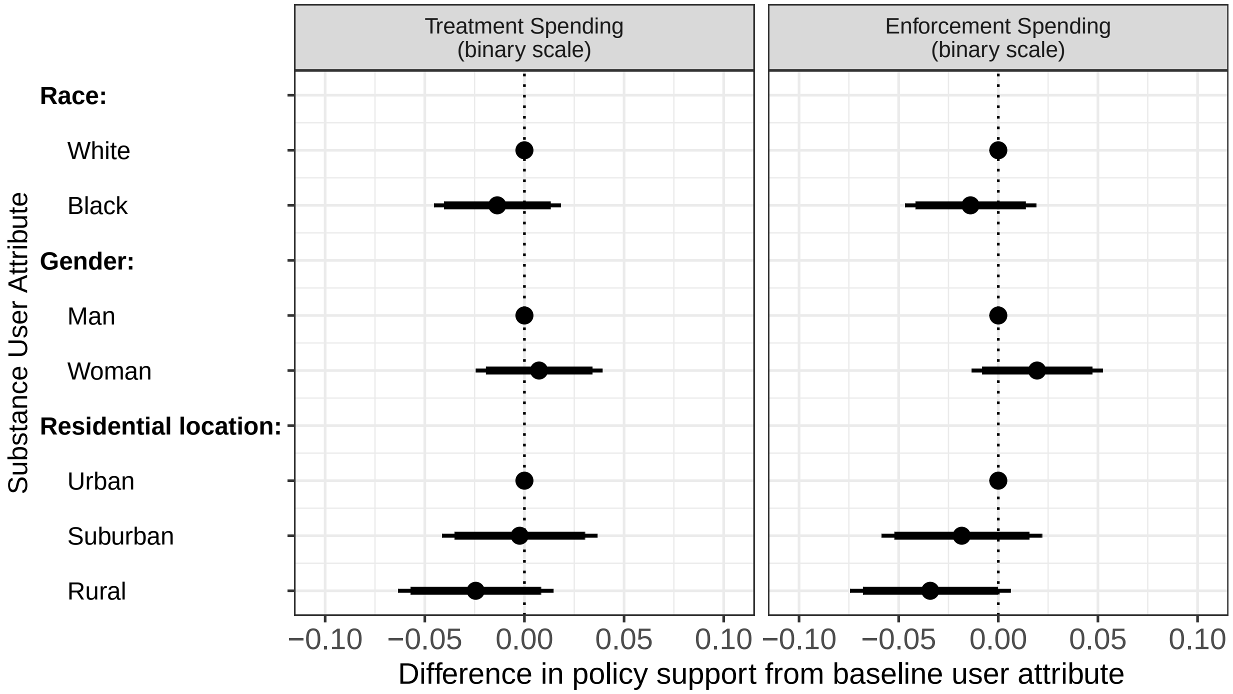

Figure G-5: Treatment effects and confidence intervals among all respondents. Points are regression coefficients and indicate the difference in levels of support for increasing policy funding between respondents in the baseline level condition (no confidence interval) compared to respondents in conditions with all other attribute levels. Lines indicate 95%-confidence intervals (thin lines) and 90%-confidence intervals (thick lines).

In addition, we also present the results exploring heterogeneity in the treatment effects of various substance user attributes using a binary outcome. In Figure G-6 we present the effect of the race treatment for both Black and white respondents.

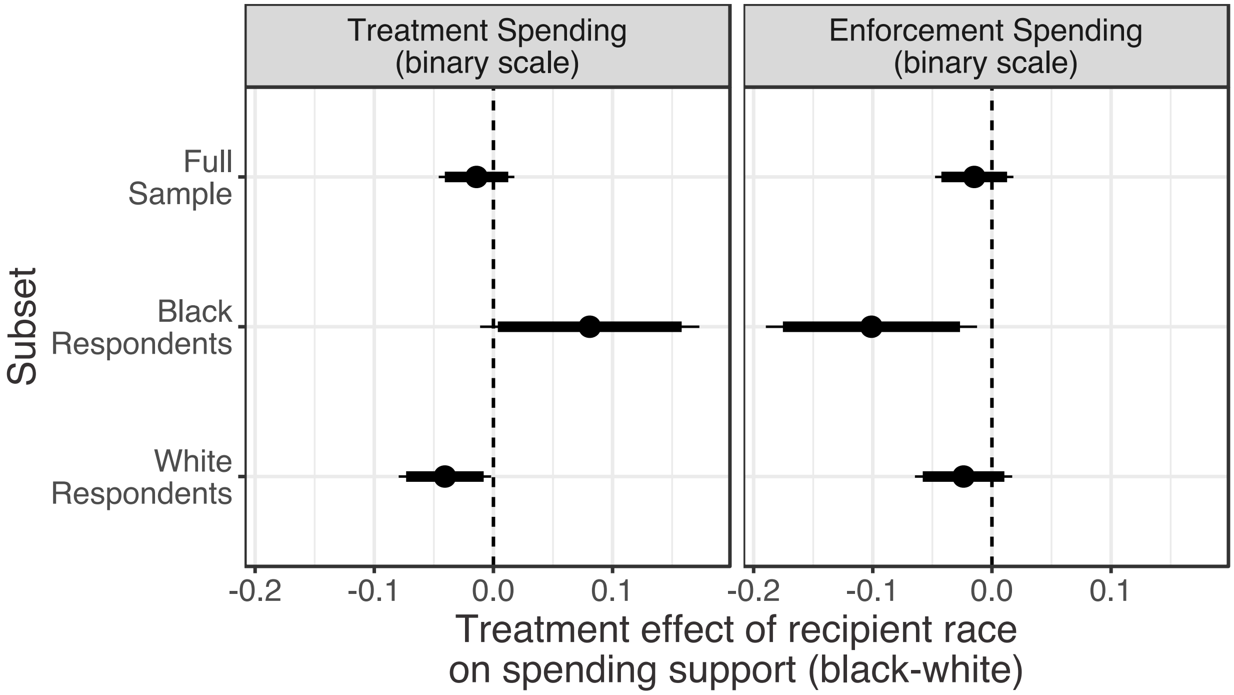

Figure G-6: Treatment effects and confidence intervals by respondent race. Points indicate the difference in levels of support for increasing policy funding between respondents who saw a Black individual profiled and a white individual profiled, with 95%-confidence intervals (thin lines) and 90%-confidence intervals (thick lines).

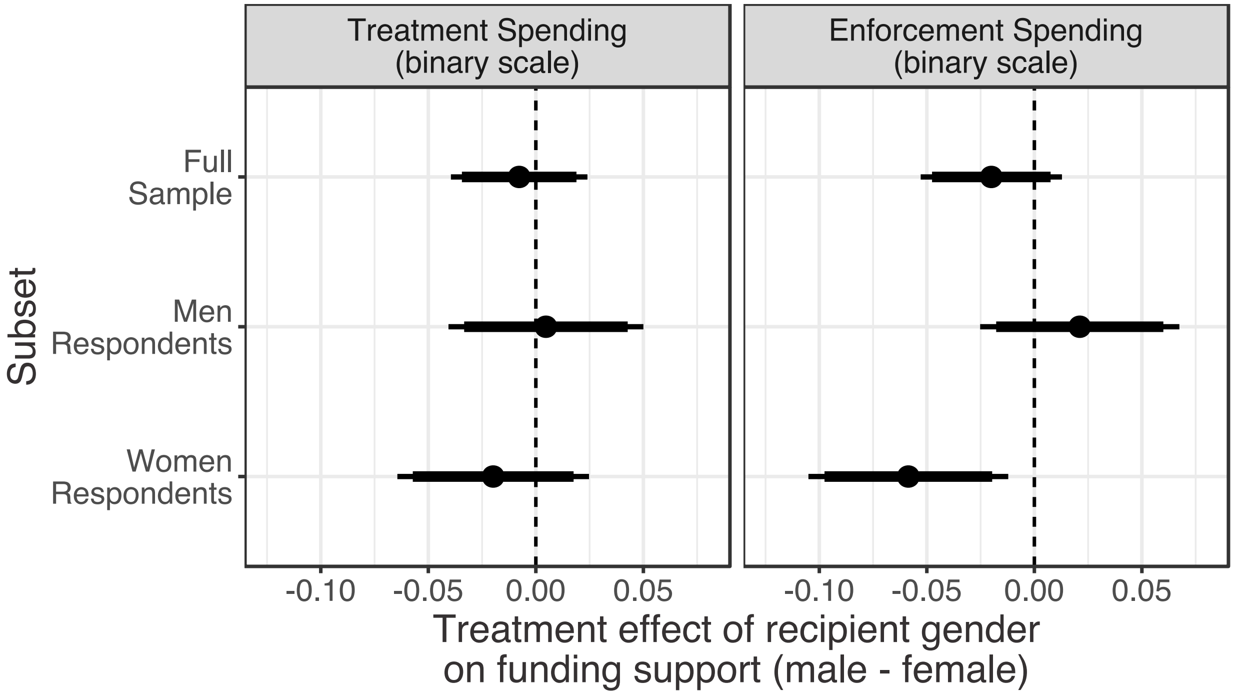

Figure G-7: Treatment effects and confidence intervals by respondent gender. Points indicate the difference in levels of support for increasing policy funding between respondents who saw a male substance user profiled and those who saw a female substance user profiled, with 95%-confidence intervals (thin lines) and 90%-confidence intervals (thick lines).

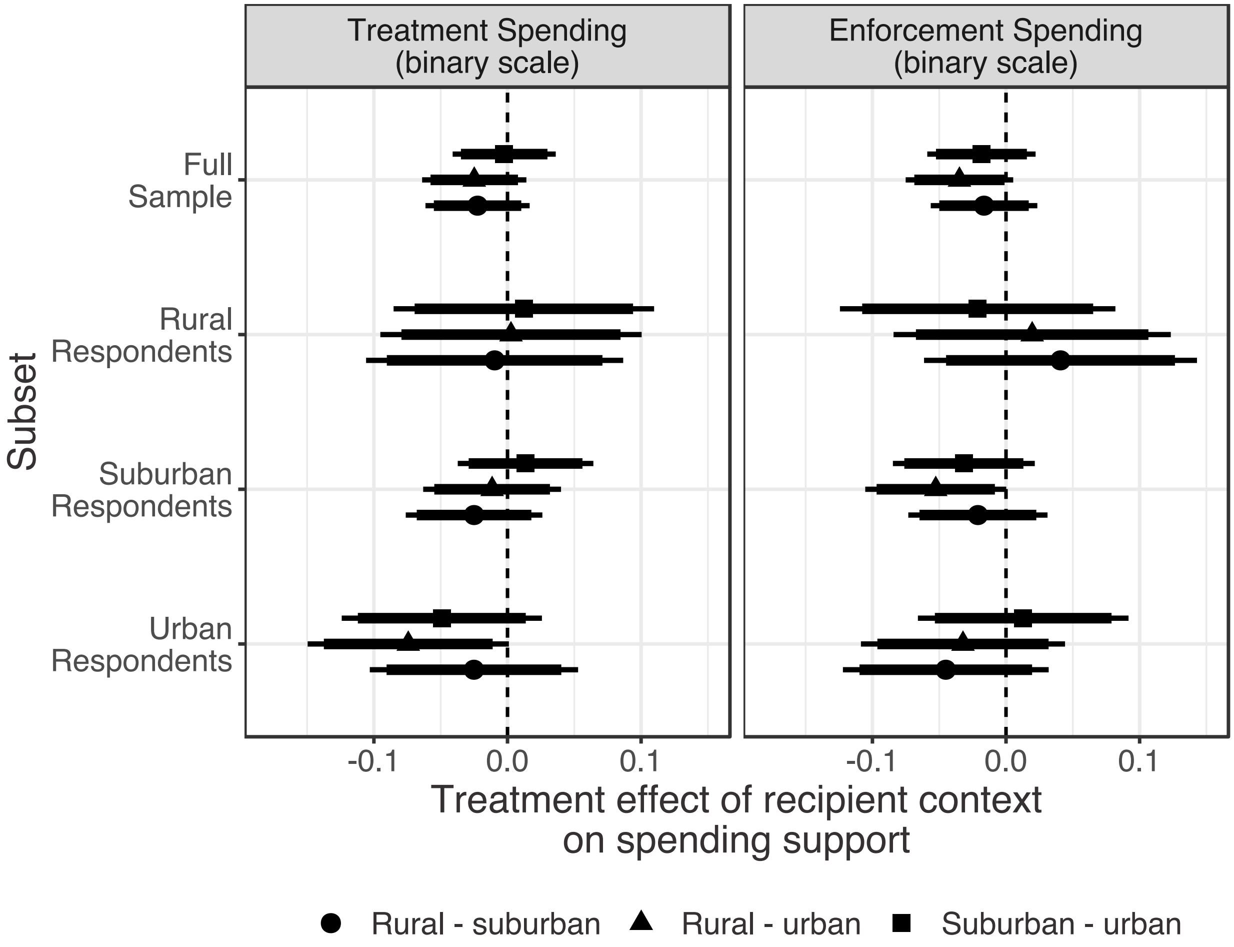

Figure G-8: Treatment effects and confidence intervals by respondent geographic context. Points indicate the difference in levels of support for increasing policy funding between respondents who saw a rural individual profiled vs. an urban individual profiled (triangles), rural vs. suburban individual (circles), or suburban vs. urban individual (squares), with 95%-confidence intervals (thin lines) and 90%-confidence intervals (thick lines).

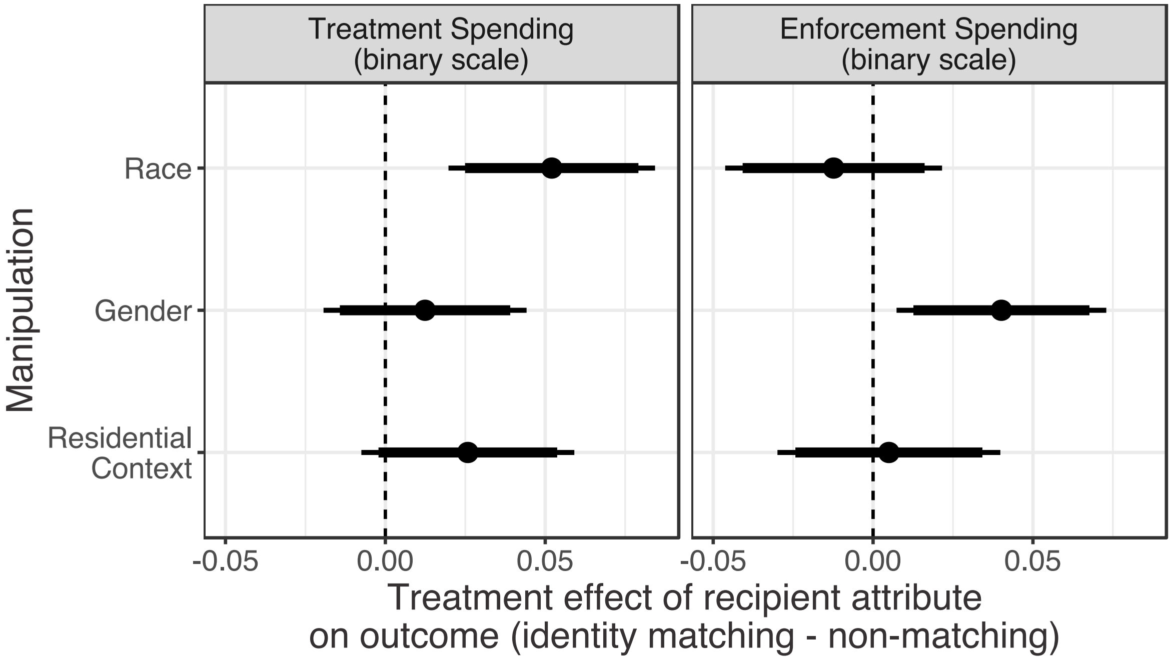

Figure G-9: Treatment effects and confidence intervals for match between respondent characteristic and substance user attributes. Points indicate the difference in each policy outcome between respondents who matched the individual profiled and those who didn't match them for each of the three identity attributes, with 95%-confidence intervals (thin lines) and 90%-confidence intervals (thick lines).

H Additional Treatments

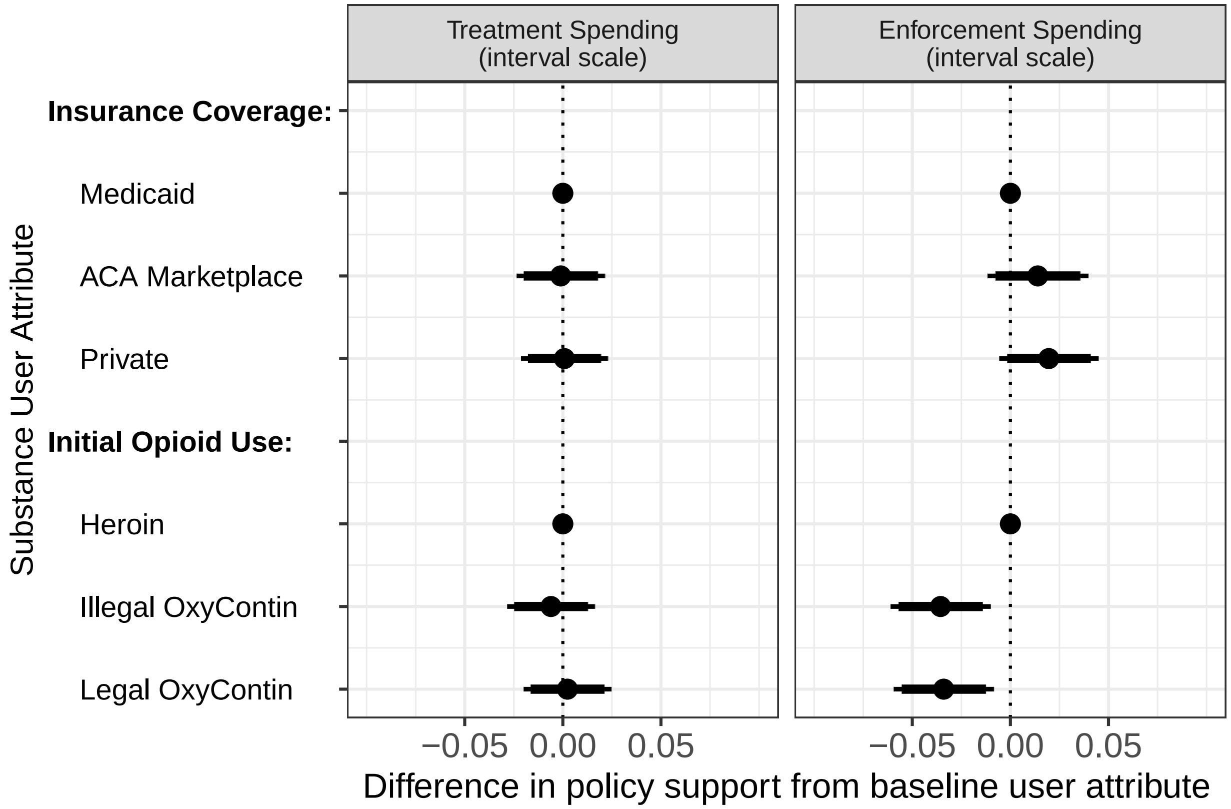

Figure H-10: Treatment effects and confidence intervals among all respondents on unit scale interval outcome. Points are regression coefficients and indicate the difference in levels of policy support between respondents in the baseline level condition (no confidence interval) compared to respondents in conditions with all other attribute levels. Lines indicate 95%-confidence intervals (thin lines) and 90%-confidence intervals (thick lines).

I Interaction Effects of Randomized Treatments

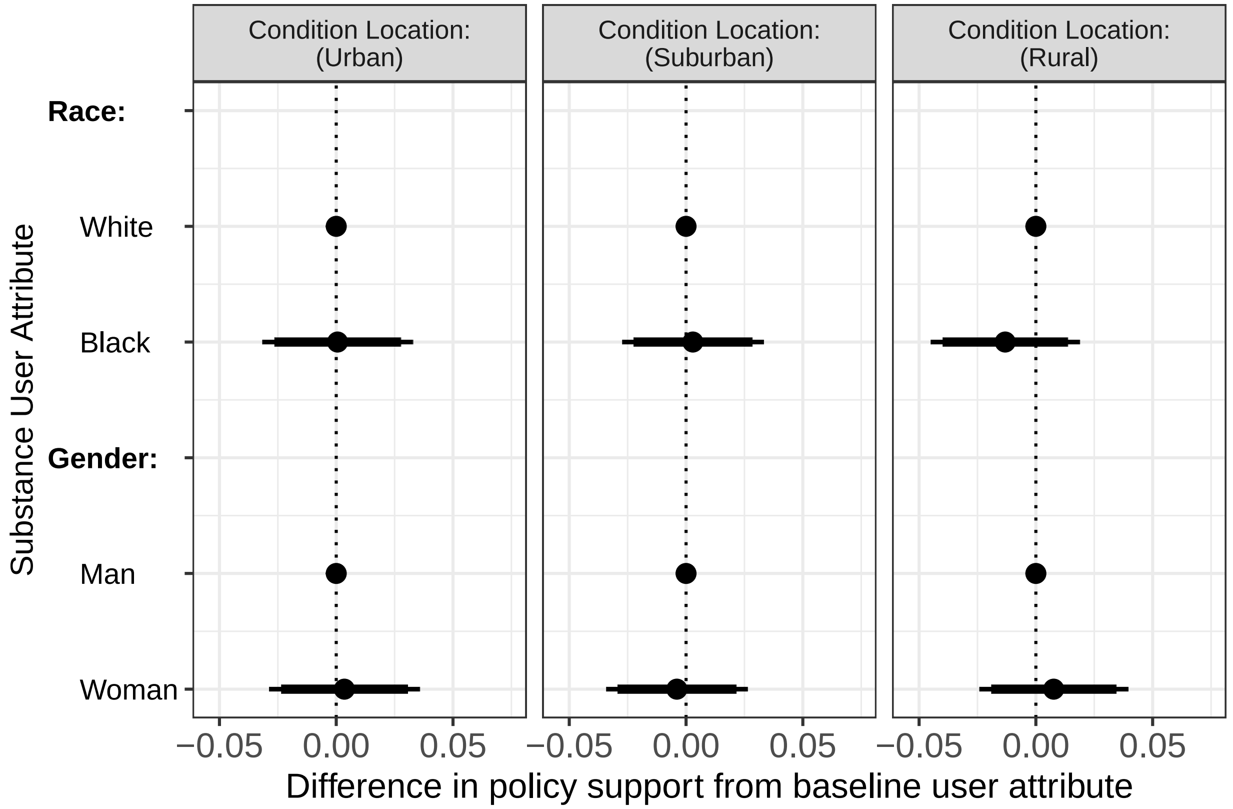

Figure I-11: Treatment effects and confidence intervals of race and gender on treatment spending by location condition on unit scale interval outcome. Points are regression coefficients and indicate the difference in levels of policy support between respondents in the baseline level condition (no confidence interval) compared to respondents in conditions with all other attribute levels. Lines indicate 95%-confidence intervals (thin lines) and 90%-confidence intervals (thick lines).

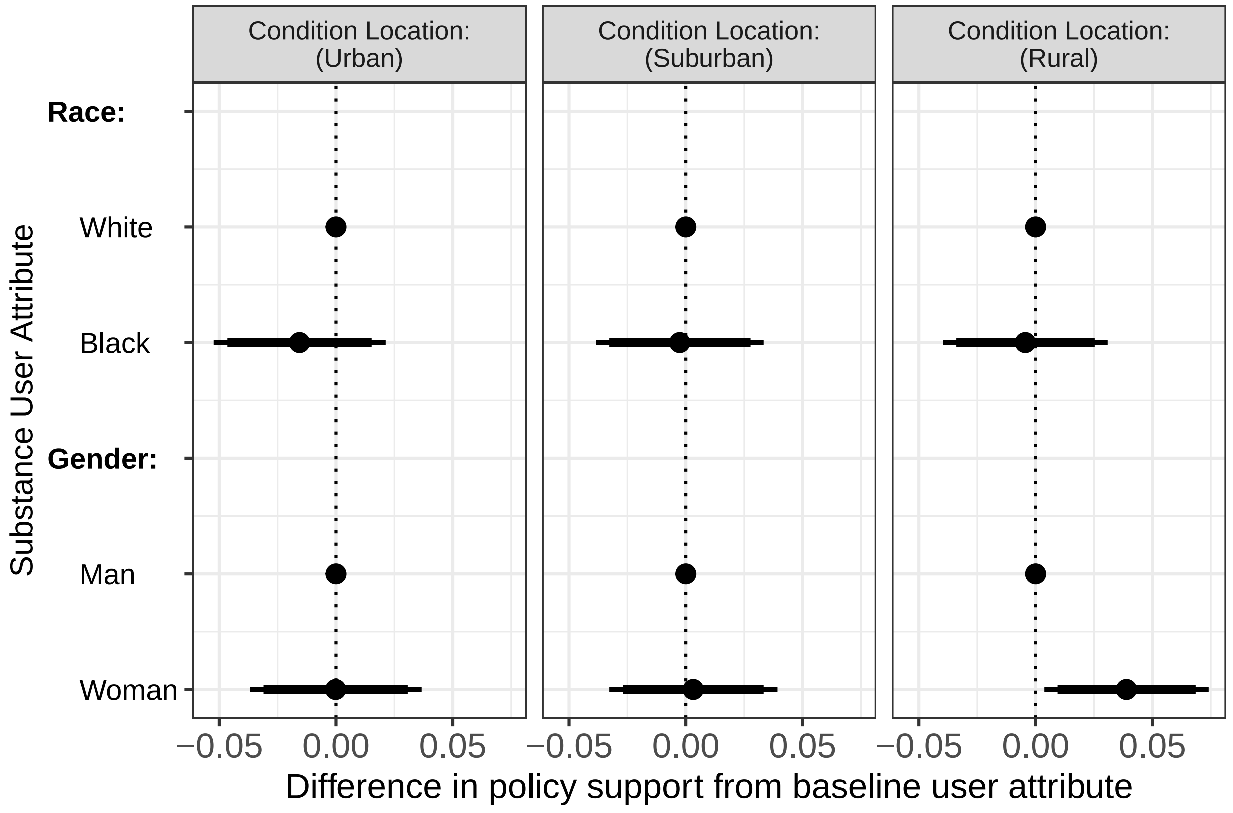

Figure I-12: Treatment effects and confidence intervals of race and gender on enforcement spending by location condition on unit scale interval outcome. Points are regression coefficients and indicate the difference in levels of policy support between respondents in the baseline level condition (no confidence interval) compared to respondents in conditions with all other attribute levels. Lines indicate 95%-confidence intervals (thin lines) and 90%-confidence intervals (thick lines).

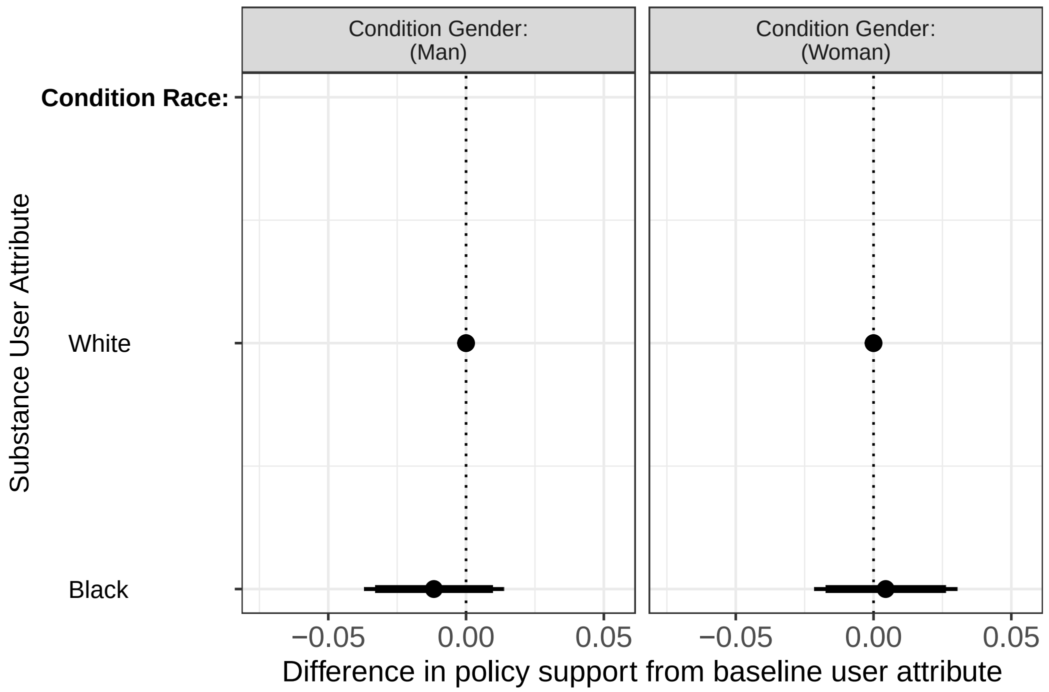

Figure I-13: Treatment effects and confidence intervals of race on treatment spending by gender condition on unit scale interval outcome. Points are regression coefficients and indicate the difference in levels of policy support between respondents in the baseline level condition (no confidence interval) compared to respondents in conditions with all other attribute levels. Lines indicate 95%-confidence intervals (thin lines) and 90%-confidence intervals (thick lines).

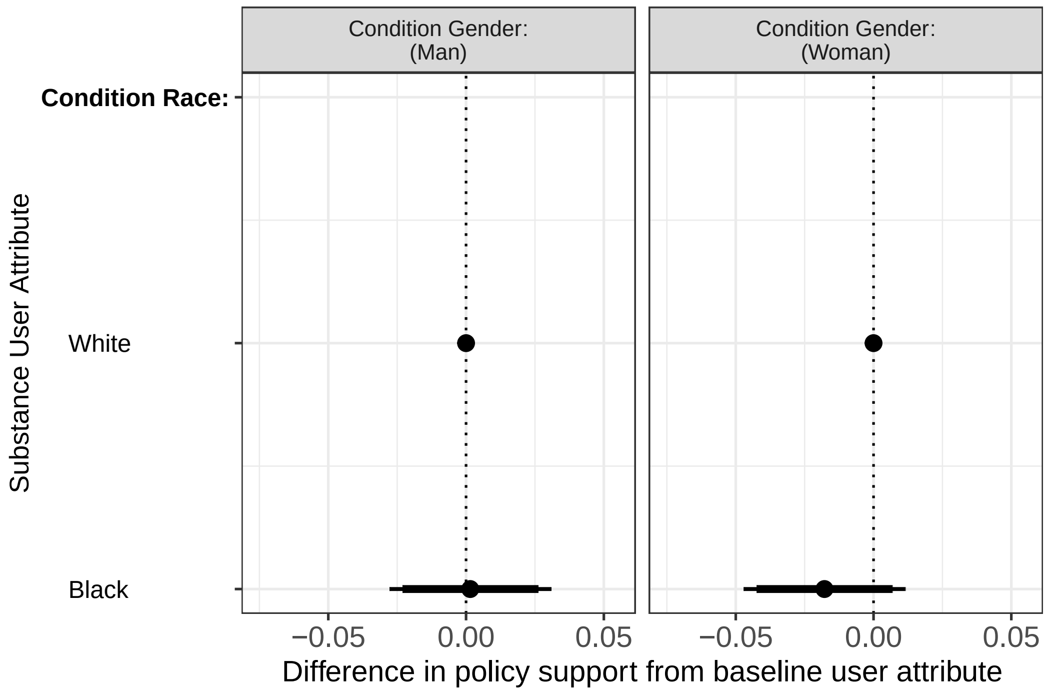

Figure I-14: Treatment effects and confidence intervals of race on enforcement spending by gender condition on unit scale interval outcome. Points are regression coefficients and indicate the difference in levels of policy support between respondents in the baseline level condition (no confidence interval) compared to respondents in conditions with all other attribute levels. Lines indicate 95%-confidence intervals (thin lines) and 90%-confidence intervals (thick lines).

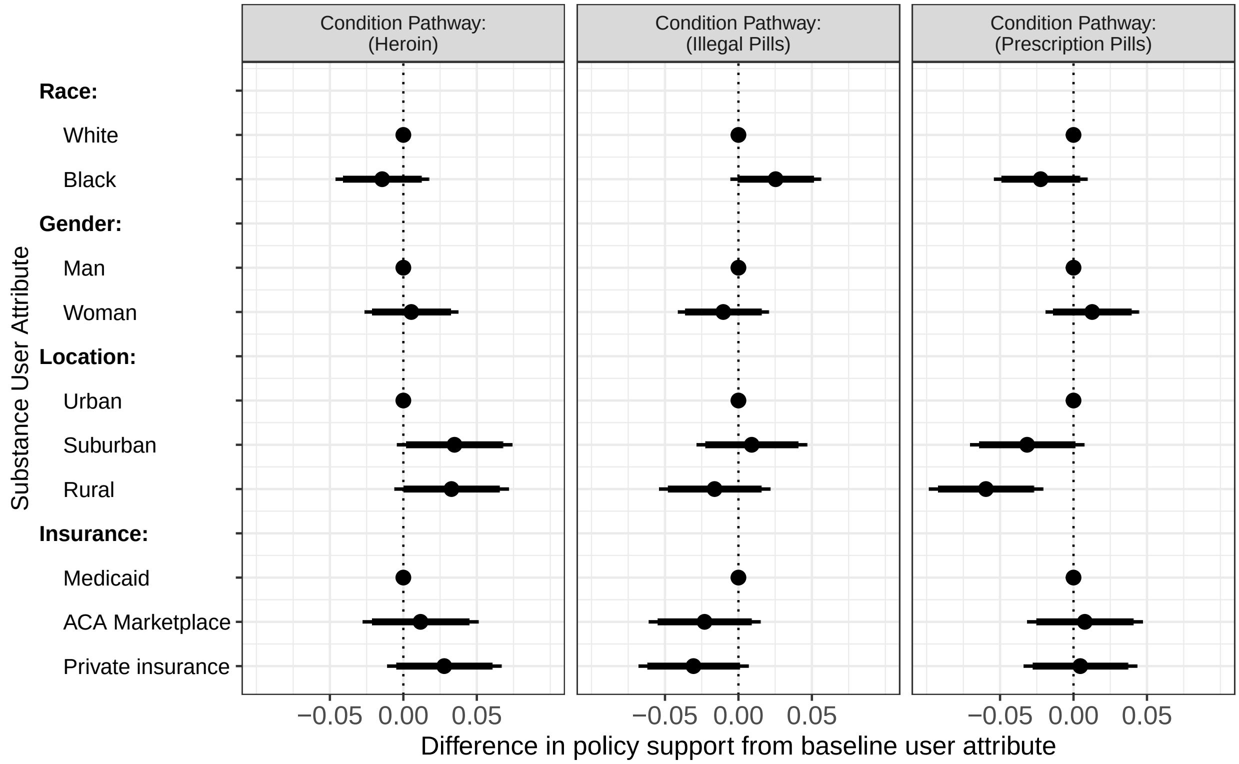

Figure I-15: Interaction of pathway to addiction and treatment variables on support for spending on treatment. Treatment effects and confidence intervals among all respondents on unit scale interval outcome. Points are regression coefficients and indicate the difference in levels of policy support between respondents in the baseline level condition (no confidence interval) compared to respondents in conditions with all other attribute levels. Lines indicate 95%-confidence intervals (thin lines) and 90%-confidence intervals (thick lines).

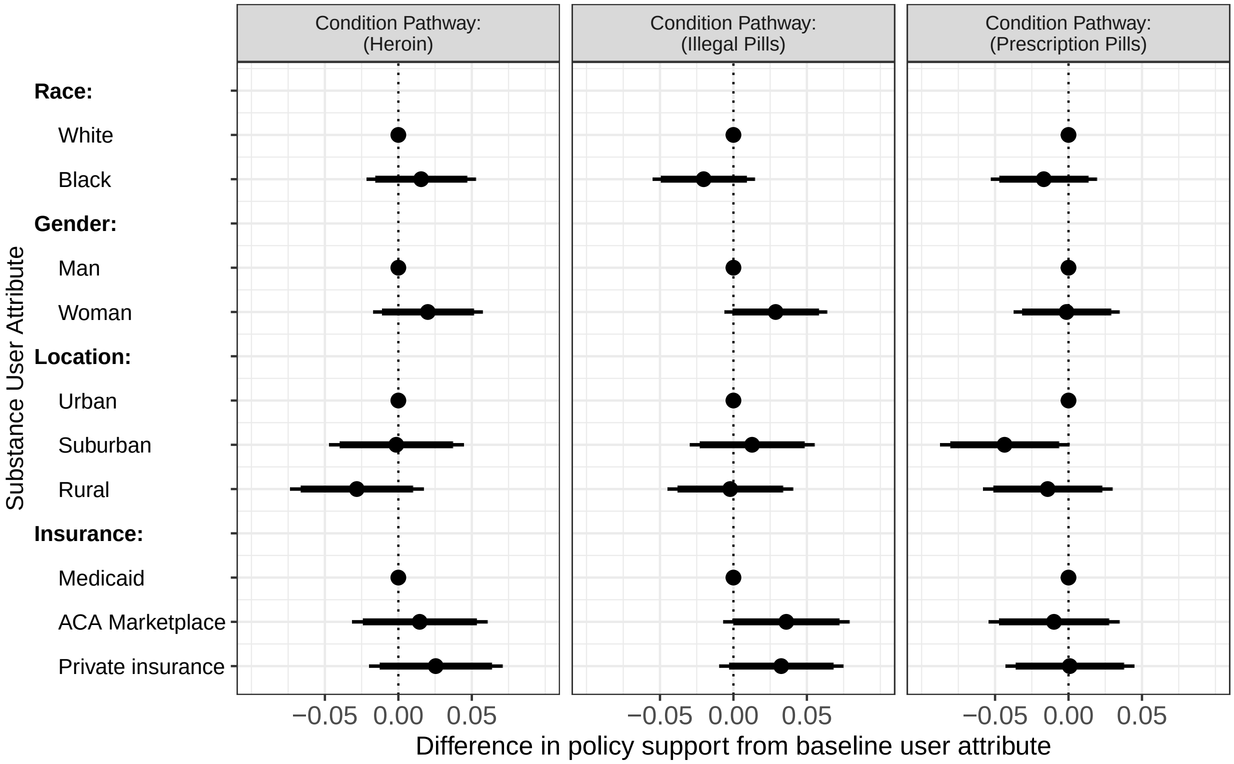

Figure I-16: Interaction of pathway to addiction and treatment variables on support for spending on enforcement. Treatment effects and confidence intervals among all respondents on unit scale interval outcome. Points are regression coefficients and indicate the difference in levels of policy support between respondents in the baseline level condition (no confidence interval) compared to respondents in conditions with all other attribute levels. Lines indicate 95%-confidence intervals (thin lines) and 90%-confidence intervals (thick lines).

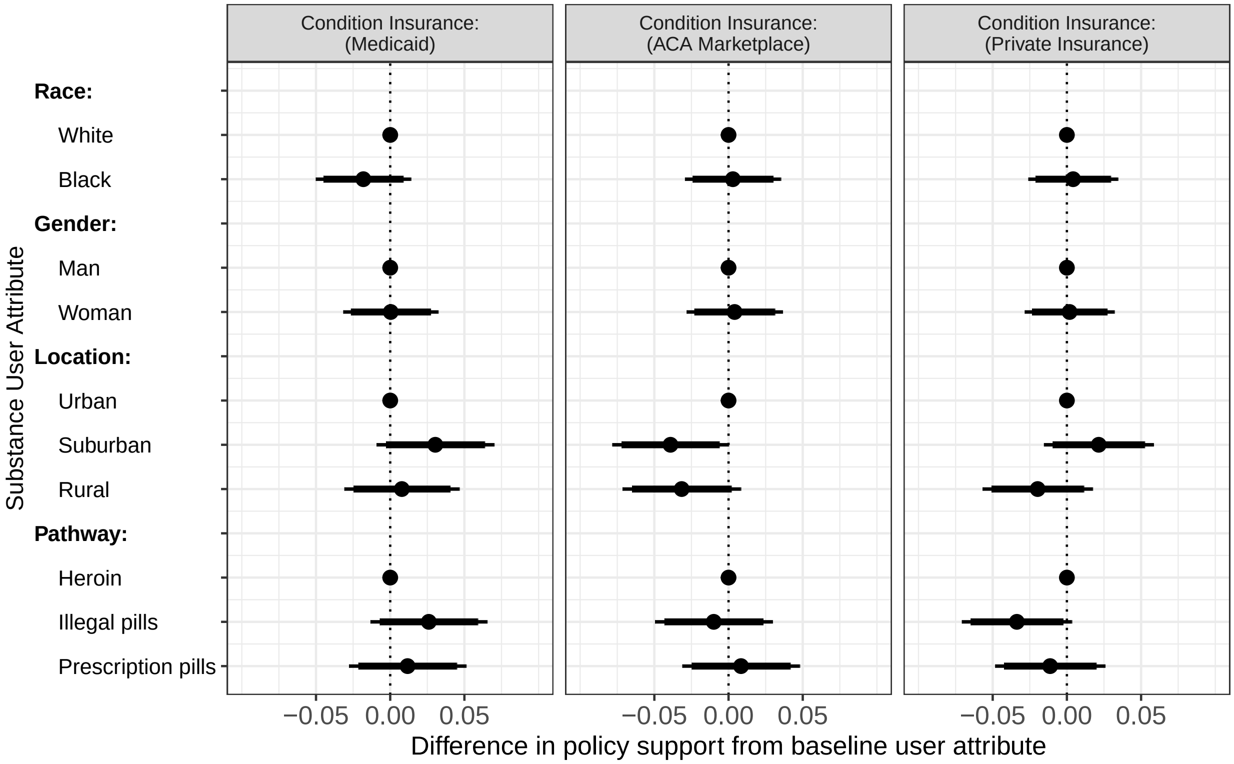

Figure I-17: Interaction of insurance and treatment variables on support for spending on treatment. Treatment effects and confidence intervals among all respondents on unit scale interval outcome. Points are regression coefficients and indicate the difference in levels of policy support between respondents in the baseline level condition (no confidence interval) compared to respondents in conditions with all other attribute levels. Lines indicate 95%-confidence intervals (thin lines) and 90%-confidence intervals (thick lines).

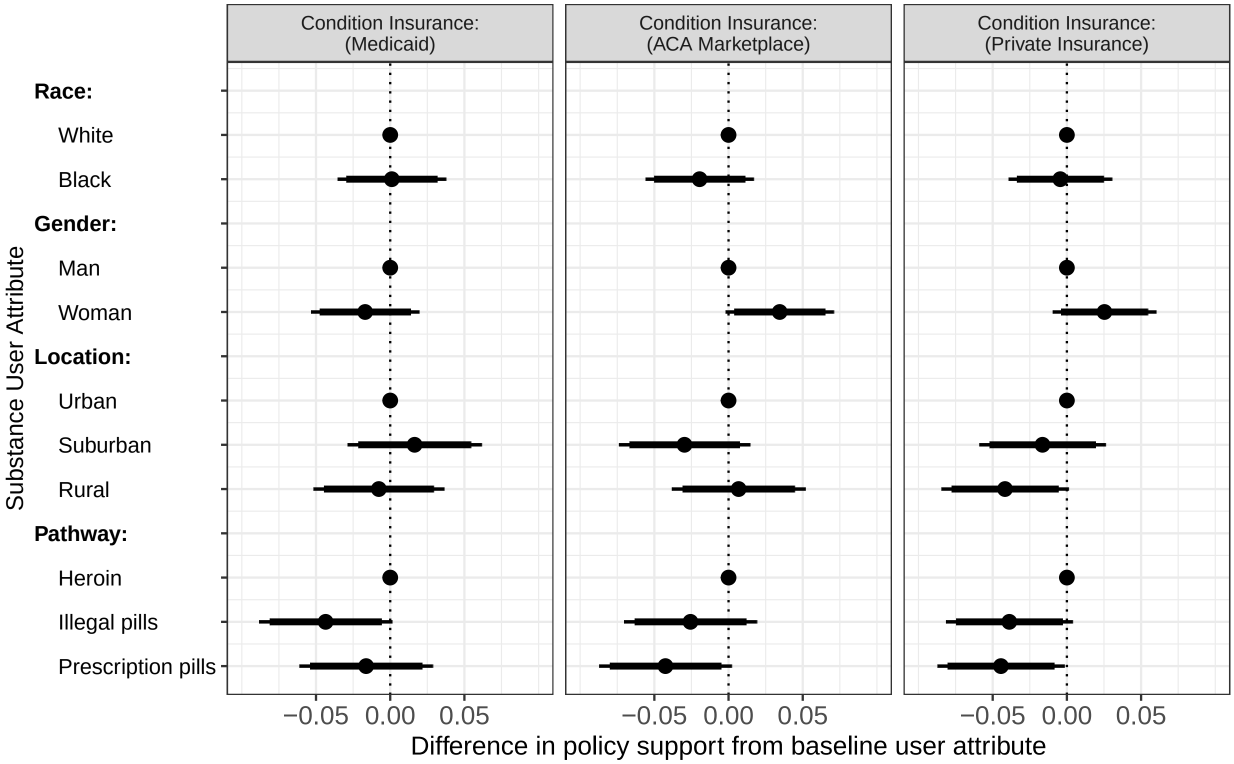

Figure I-18: Interaction of insurance and treatment variables on support for spending on enforcement. Treatment effects and confidence intervals among all respondents on unit scale interval outcome. Points are regression coefficients and indicate the difference in levels of policy support between respondents in the baseline level condition (no confidence interval) compared to respondents in conditions with all other attribute levels. Lines indicate 95%-confidence intervals (thin lines) and 90%-confidence intervals (thick lines).

J Heterogeneity in the Effects by Respondent Partisanship

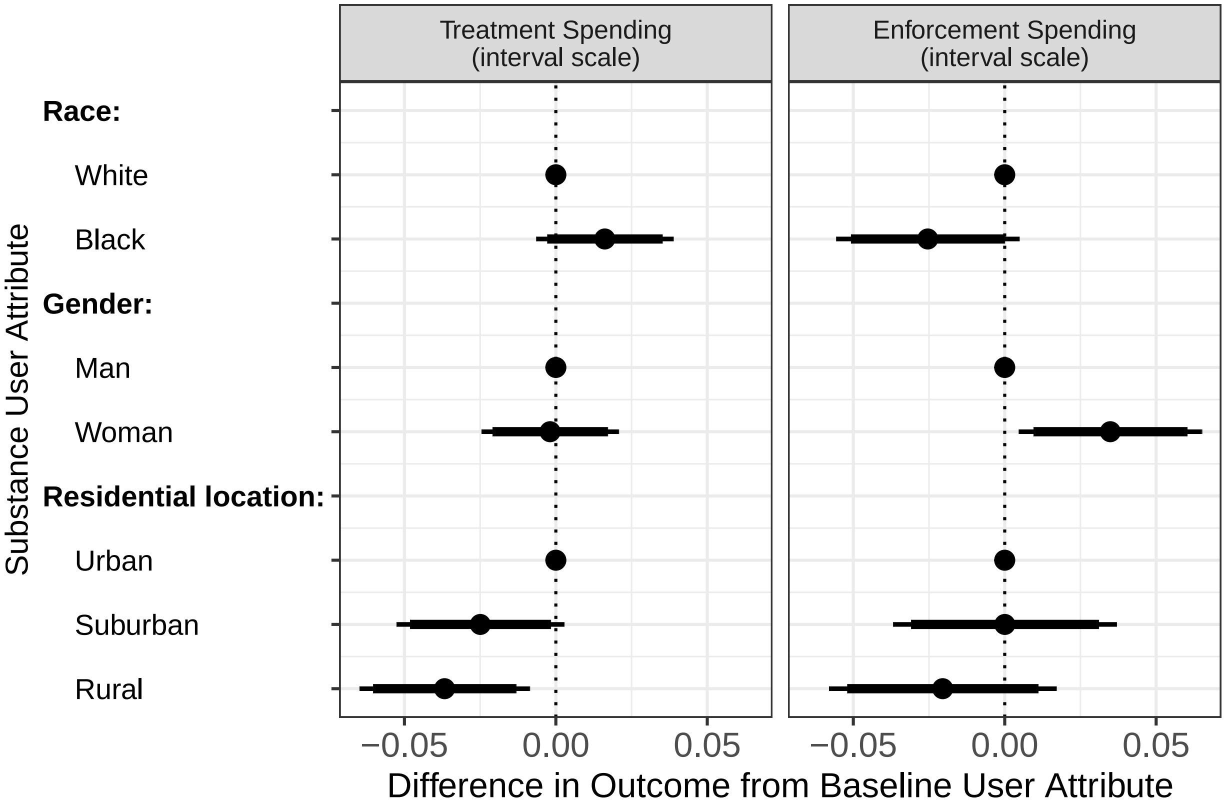

Figure J-19: Treatment effects and confidence intervals among Democratic respondents on unit scale interval outcome. Points are regression coefficients and indicate the difference in levels of policy support between respondents in the baseline level condition (no confidence interval) compared to respondents in conditions with all other attribute levels. Lines indicate 95%-confidence intervals (thin lines) and 90%-confidence intervals (thick lines).

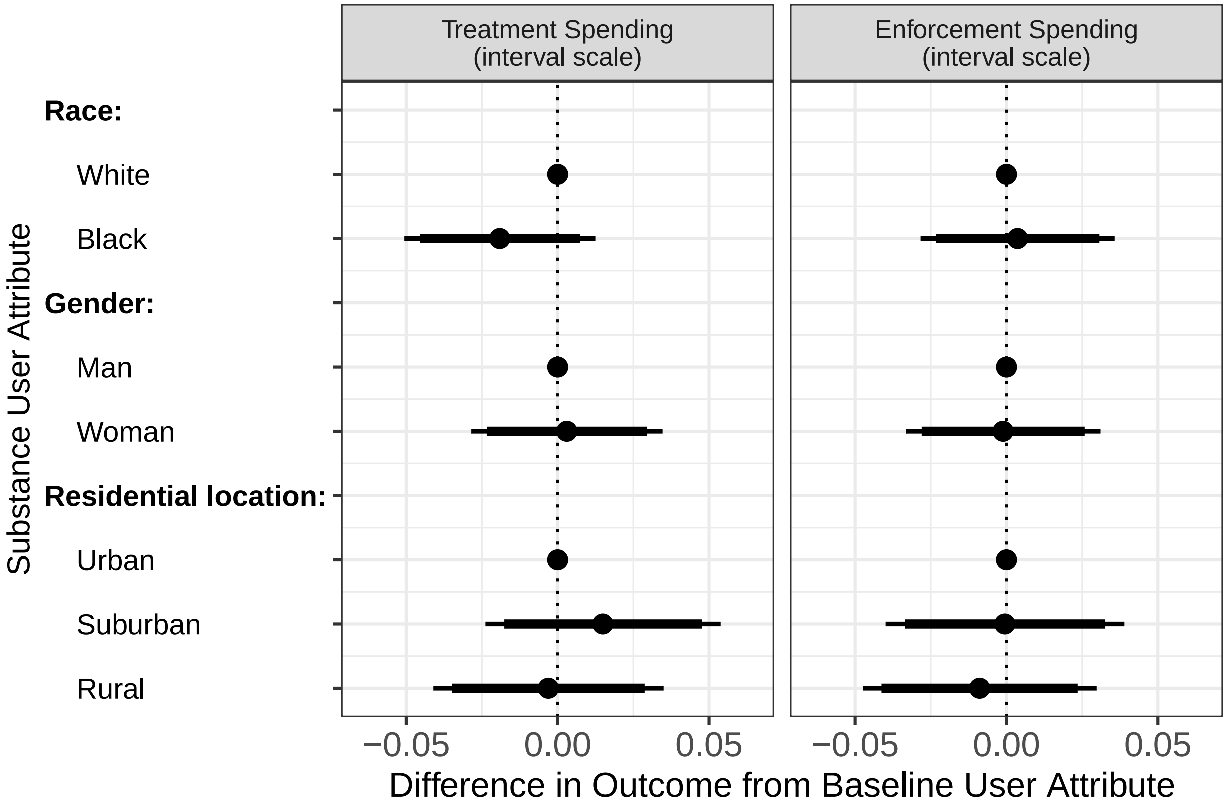

Figure J-20: Treatment effects and confidence intervals among Republican respondents on unit scale interval outcome. Points are regression coefficients and indicate the difference in levels of policy support between respondents in the baseline level condition (no confidence interval) compared to respondents in conditions with all other attribute levels. Lines indicate 95%-confidence intervals (thin lines) and 90%-confidence intervals (thick lines).

K Multiple Hypothesis Testing Corrections

To account for multiple hypothesis testing, we estimate a false discovery rate (FDR) — the rate that features called significant are truly null — for every hypothesis test in our analysis. The FDR has been shown to be uniformly more powerful that the traditional Bonferroni method (Storey and Tibshirani, 2003). In Table K-2, we report the original p-value and corrected q-value for each hypothesis test.

For example, Figure 6 shows that sharing a racial identity with the profiled substance user causes a respondent to increase their support for funding addiction treatment programs by 3 percentage points. The estimate's p-value of < 0.01 indicates that there is a less than 1 percent chance of observing as great (or greater) test statistic under the assumption that the null hypothesis is correct. In a similar sense, the estimate's q-value of 0.05 allows us to say that 5 percent of the test statistics that are as extreme as the "Race (matching) on treatment" hypothesis are false positives. This makes increases our confidence that the primacy of shared racial identity in the shaping opioid policy is not the result of multiple hypothesis testing.

Figure	Spending DV	Hypothesis	P-Value	Q-Value
Figure 2	Treatment	Race	0.72	0.98
Figure 2	Treatment	Gender	0.78	1.00
Figure 2	Treatment	Context (suburban)	0.71	0.97
Figure 2	Treatment	Context (rural)	0.20	0.69
Figure 2	Enforcement	Race	0.46	0.81
Figure 2	Enforcement	Gender	0.19	0.68
Figure 2	Enforcement	Context (suburban)	0.39	0.80
Figure 2	Enforcement	Context (rural)	0.25	0.71
Figure 3	Treatment	Race by respondent (Black)	0.11	0.55
Figure 3	Treatment	Race by respondent (white)	0.06	0.51
Figure 3	Enforcement	Race by respondent (Black)	0.03	0.48
Figure 3	Enforcement	Race by respondent (white)	0.42	0.80
Figure 4	Treatment	Gender by respondent (man)	0.71	0.97
Figure 4	Treatment	Gender by respondent (woman)	0.43	0.80
Figure 4	Enforcement	Gender by respondent (man)	0.77	1.00
Figure 4	Enforcement	Gender by respondent (woman)	0.11	0.55
Figure 5	Treatment	Context (sub. v. urban) by resp. (rural)	0.22	0.71
Figure 5	Treatment	Context (rural v. urban) by resp. (rural)	0.92	1.00
Figure 5	Treatment	Context (rural v. sub.) by resp. (rural)	0.22	0.71
Figure 5	Treatment	Context (sub. v. urban) by resp. (sub.)	0.91	1.00
Figure 5	Treatment	Context (rural v. urban) by resp. (sub.)	0.50	0.86
Figure 5	Treatment	Context (rural v. sub.) by resp. (sub.)	0.57	0.88
Figure 5	Treatment	Context (sub. v. urban) by resp. (urban)	0.86	1.00
Figure 5	Treatment	Context (rural v. urban) by resp. (urban)	0.11	0.55
Figure 5	Treatment	Context (rural v. sub.) by resp. (urban)	0.13	0.57
Figure 5	Enforcement	Context (sub. v. urban) by resp. (rural)	0.93	1.00
Figure 5	Enforcement	Context (rural v. urban) by resp. (rural)	0.76	1.00
Figure 5	Enforcement	Context (rural v. sub.) by resp. (rural)	0.69	0.97
Figure 5	Enforcement	Context (sub. v. urban) by resp. (sub.)	0.26	0.71
Figure 5	Enforcement	Context (rural v. urban) by resp. (sub.)	0.25	0.71
Figure 5	Enforcement	Context (rural v. sub.) by resp. (sub.)	0.99	1.00
Figure 5	Enforcement	Context (sub. v. urban) by resp. (urban)	0.97	1.00
Figure 5	Enforcement	Context (rural v. urban) by resp. (urban)	0.35	0.80
Figure 5	Enforcement	Context (rural v. sub.) by resp. (urban)	0.34	0.80
Figure 6	Treatment	Race (matching)	0.00	0.05
Figure 6	Treatment	Gender (matching)	0.41	0.80
Figure 6	Treatment	Context (matching)	0.66	0.96
Figure 6	Enforcement	Race (matching)	0.15	0.61
Figure 6	Enforcement	Gender (matching)	0.38	0.80
Figure 6	Enforcement	Context (matching	0.97	1.00
Figure 7	Blame	Illegal pills	0.00	0.08
Figure 7	Blame	Legal pills	0.00	0.00
Figure 8	Treatment	Race (matching) x Heroin	0.37	0.80

Figure 8	Treatment	Gender (matching) x Heroin	0.65	0.96
Figure 8	Treatment	Context (matching) x Heroin	0.92	1.00
Figure 8	Enforcement	Race (matching) x Heroin	0.00	0.04
Figure 8	Enforcement	Gender (matching) x Heroin	0.07	0.52
Figure 8	Enforcement	Context (matching) x Heroin	0.48	0.83
Figure 8	Treatment	Race (matching) x Legal pills	0.05	0.51
Figure 8	Treatment	Gender (matching) x Legal pills	0.37	0.80
Figure 8	Treatment	Context (matching) x Legal pills	0.27	0.71
Figure 8	Enforcement	Race (matching) x Legal pills	0.87	1.00
Figure 8	Enforcement	Gender (matching) x Legal pills	0.51	0.86
Figure 8	Enforcement	Context (matching) x Legal pills	0.28	0.71
Figure G5	Treatment	Race	0.40	0.80
Figure G5	Treatment	Gender	0.65	0.96
Figure G5	Treatment	Context (suburban)	0.90	1.00
Figure G5	Treatment	Context (rural)	0.22	0.71
Figure G5	Enforcement	Race	0.40	0.80
Figure G5	Enforcement	Gender	0.25	0.71
Figure G5	Enforcement	Context (suburban)	0.37	0.80
Figure G5	Enforcement	Context (rural)	0.10	0.55
Figure G6	Treatment	Race by respondent (Black)	0.08	0.54
Figure G6	Treatment	Race by respondent (white)	0.04	0.51
Figure G6	Enforcement	Race by respondent (Black)	0.03	0.45
Figure G6	Enforcement	Race by respondent (white)	0.25	0.71
Figure G7	Treatment	Gender by respondent (man)	0.84	1.00
Figure G7	Treatment	Gender by respondent (woman)	0.38	0.80
Figure G7	Enforcement	Gender by respondent (man)	0.37	0.80
Figure G7	Enforcement	Gender by respondent (woman)	0.01	0.34
Figure G8	Treatment	Context (sub. v. urban) by resp. (rural)	0.81	1.00
Figure G8	Treatment	Context (rural v. urban) by resp. (rural)	0.96	1.00
Figure G8	Treatment	Context (rural v. sub.) by resp. (rural)	0.84	1.00
Figure G8	Treatment	Context (sub. v. urban) by resp. (sub.)	0.60	0.92
Figure G8	Treatment	Context (rural v. urban) by resp. (sub.)	0.66	0.96
Figure G8	Treatment	Context (rural v. sub.) by resp. (sub.)	0.34	0.80
Figure G8	Treatment	Context (sub. v. urban) by resp. (urban)	0.20	0.69
Figure G8	Treatment	Context (rural v. urban) by resp. (urban)	0.05	0.51
Figure G8	Treatment	Context (rural v. sub.) by resp. (urban)	0.53	0.87
Figure G8	Enforcement	Context (sub. v. urban) by resp. (rural)	0.69	0.97
Figure G8	Enforcement	Context (rural v. urban) by resp. (rural)	0.71	0.97
Figure G8	Enforcement	Context (rural v. sub.) by resp. (rural)	0.43	0.80
Figure G8	Enforcement	Context (sub. v. urban) by resp. (sub.)	0.24	0.71
Figure G8	Enforcement	Context (rural v. urban) by resp. (sub.)	0.05	0.51
Figure G8	Enforcement	Context (rural v. sub.) by resp. (sub.)	0.43	0.80
Figure G8	Enforcement	Context (sub. v. urban) by resp. (urban)	0.75	1.00
Figure G8	Enforcement	Context (rural v. urban) by resp. (urban)	0.41	0.80

Figure G8	Enforcement	Context (rural v. sub.) by resp. (urban)	0.25	0.71
Figure G9	Treatment	Race (matching)	0.00	0.08
Figure G9	Treatment	Gender (matching)	0.44	0.80
Figure G9	Treatment	Context (matching)	0.13	0.57
Figure G9	Enforcement	Race (matching)	0.48	0.83
Figure G9	Enforcement	Gender (matching)	0.02	0.36
Figure G9	Enforcement	Context (matching)	0.78	1.00
Figure H10	Treatment	Insurance (ACA)	0.92	1.00
Figure H10	Treatment	Insurance (Private)	0.95	1.00
Figure H10	Treatment	Pathway (Illegal pills)	0.59	0.92
Figure H10	Treatment	Pathway (Legal pills)	0.84	1.00
Figure H10	Enforcement	Insurance (ACA)	0.29	0.71
Figure H10	Enforcement	Insurance (Private)	0.13	0.57
Figure H10	Enforcement	Pathway (Illegal pills)	0.01	0.22
Figure H10	Enforcement	Pathway (Legal pills)	0.01	0.28
Figure I11	Treatment	Race x Urban	0.97	1.00
Figure I11	Treatment	Gender x Urban	0.83	1.00
Figure I11	Treatment	Race x Suburban	0.85	1.00
Figure I11	Treatment	Gender x Suburban	0.80	1.00
Figure I11	Treatment	Race x Rural	0.42	0.80
Figure I11	Treatment	Gender x Rural	0.64	0.96
Figure I12	Enforcement	Race x Urban	0.41	0.80
Figure I12	Enforcement	Gender x Urban	0.99	1.00
Figure I12	Enforcement	Race x Suburban	0.89	1.00
Figure I12	Enforcement	Gender x Suburban	0.86	1.00
Figure I12	Enforcement	Race x Rural	0.81	1.00
Figure I12	Enforcement	Gender x Rural	0.03	0.47
Figure I13	Treatment	Race x Man	0.37	0.80
Figure I13	Treatment	Race x Woman	0.74	0.99
Figure I14	Enforcement	Race x Man	0.92	1.00
Figure I14	Enforcement	Race x Woman	0.23	0.71
Figure I15	Treatment	Race x Heroin	0.37	0.80
Figure I15	Treatment	Gender x Heroin	0.74	0.99
Figure I15	Treatment	Context (suburban) x Heroin	0.08	0.54
Figure I15	Treatment	Context (rural) x Heroin	0.10	0.55
Figure I15	Treatment	Insurance (ACA) x Heroin	0.56	0.88
Figure I15	Treatment	Insurance (private) x Heroin	0.16	0.63
Figure I15	Treatment	Race x Illegal pills	0.11	0.55
Figure I15	Treatment	Gender x Illegal pills	0.51	0.86
Figure I15	Treatment	Context (suburban) x Illegal pills	0.64	0.96
Figure I15	Treatment	Context (rural) x Illegal pills	0.40	0.80
Figure I15	Treatment	Insurance (ACA) x Illegal pills	0.23	0.71
Figure I15	Treatment	Insurance (private) x Illegal pills	0.11	0.55
Figure I15	Treatment	Race x Legal pills	0.17	0.64

Figure I15	Treatment	Gender x Legal pills	0.43	0.80
Figure I15	Treatment	Context (suburban) x Legal pills	0.11	0.55
Figure I15	Treatment	Context (rural) x Legal pills	0.00	0.11
Figure I15	Treatment	Insurance (ACA) x Legal pills	0.70	0.97
Figure I15	Treatment	Insurance (private) x Legal pills	0.81	1.00
Figure I16	Enforcement	Race x Heroin	0.41	0.80
Figure I16	Enforcement	Gender x Heroin	0.29	0.71
Figure I16	Enforcement	Context (suburban) x Heroin	0.95	1.00
Figure I16	Enforcement	Context (rural) x Heroin	0.22	0.71
Figure I16	Enforcement	Insurance (ACA) x Heroin	0.54	0.87
Figure I16	Enforcement	Insurance (private) x Heroin	0.27	0.71
Figure I16	Enforcement	Race x Illegal pills	0.25	0.71
Figure I16	Enforcement	Gender x Illegal pills	0.11	0.55
Figure I16	Enforcement	Context (suburban) x Illegal pills	0.56	0.88
Figure I16	Enforcement	Context (rural) x Illegal pills	0.92	1.00
Figure I16	Enforcement	Insurance (ACA) x Illegal pills	0.10	0.55
Figure I16	Enforcement	Insurance (private) x Illegal pills	0.13	0.57
Figure I16	Enforcement	Race x Legal pills	0.36	0.80
Figure I16	Enforcement	Gender x Legal pills	0.94	1.00
Figure I16	Enforcement	Context (suburban) x Legal pills	0.05	0.51
Figure I16	Enforcement	Context (rural) x Legal pills	0.53	0.87
Figure I16	Enforcement	Insurance (ACA) x Legal pills	0.66	0.96
Figure I16	Enforcement	Insurance (private) x Legal pills	0.97	1.00
Figure I17	Treatment	Race x Medicaid	0.27	0.71
Figure I17	Treatment	Gender x Medicaid	0.98	1.00
Figure I17	Treatment	Context (suburban) x Medicaid	0.13	0.57
Figure I17	Treatment	Context (rural) x Medicaid	0.69	0.97
Figure I17	Treatment	Pathway (illegal pills) x Medicaid	0.20	0.69
Figure I17	Treatment	Pathway (legal pills) x Medicaid	0.56	0.88
Figure I17	Treatment	Race x ACA	0.86	1.00
Figure I17	Treatment	Gender x ACA	0.80	1.00
Figure I17	Treatment	Context (suburban) x ACA	0.05	0.51
Figure I17	Treatment	Context (rural) x ACA	0.12	0.57
Figure I17	Treatment	Pathway (illegal pills) x ACA	0.62	0.94
Figure I17	Treatment	Pathway (legal pills) x ACA	0.68	0.97
Figure I17	Treatment	Race x Private	0.78	1.00
Figure I17	Treatment	Gender x Private	0.91	1.00
Figure I17	Treatment	Context (suburban) x Private	0.26	0.71
Figure I17	Treatment	Context (rural) x Private	0.30	0.71
Figure I17	Treatment	Pathway (illegal pills) x Private	0.07	0.54
Figure I17	Treatment	Pathway (legal pills) x Private	0.55	0.88
Figure I18	Enforcement	Race x Medicaid	0.95	1.00
Figure I18	Enforcement	Gender x Medicaid	0.36	0.80
Figure I18	Enforcement	Context (suburban) x Medicaid	0.48	0.83

Figure I18	Enforcement	Context (rural) x Medicaid	0.73	0.99
Figure I18	Enforcement	Pathway (illegal pills) x Medicaid	0.06	0.51
Figure I18	Enforcement	Pathway (legal pills) x Medicaid	0.48	0.83
Figure I18	Enforcement	Race x ACA	0.29	0.71
Figure I18	Enforcement	Gender x ACA	0.06	0.52
Figure I18	Enforcement	Context (suburban) x ACA	0.19	0.68
Figure I18	Enforcement	Context (rural) x ACA	0.77	1.00
Figure I18	Enforcement	Pathway (illegal pills) x ACA	0.26	0.71
Figure I18	Enforcement	Pathway (legal pills) x ACA	0.06	0.52
Figure I18	Enforcement	Race x Private	0.80	1.00
Figure I18	Enforcement	Gender x Private	0.15	0.62
Figure I18	Enforcement	Context (suburban) x Private	0.45	0.80
Figure I18	Enforcement	Context (rural) x Private	0.06	0.51
Figure I18	Enforcement	Pathway (illegal pills) x Private	0.08	0.54
Figure I18	Enforcement	Pathway (legal pills) x Private	0.04	0.51
Figure J19	Treatment	Race, Democrats	0.16	0.63
Figure J19	Treatment	Gender, Democrats	0.87	1.00
Figure J19	Treatment	Context (suburban), Democrats	0.08	0.54
Figure J19	Treatment	Context (rural), Democrats	0.01	0.29
Figure J19	Enforcement	Race, Democrats	0.10	0.55
Figure J19	Enforcement	Gender, Democrats	0.02	0.45
Figure J19	Enforcement	Context (suburban), Democrats	1.00	1.00
Figure J19	Enforcement	Context (rural), Democrats	0.28	0.71
Figure J20	Treatment	Race, Republicans	0.23	0.71
Figure J20	Treatment	Gender, Republicans	0.85	1.00
Figure J20	Treatment	Context (suburban), Republicans	0.45	0.80
Figure J20	Treatment	Context (rural), Republicans	0.87	1.00
Figure J20	Enforcement	Race, Republicans	0.82	1.00
Figure J20	Enforcement	Gender, Republicans	0.94	1.00
Figure J20	Enforcement	Context (suburban), Republicans	0.98	1.00
Figure J20	Enforcement	Context (rural), Republicans	0.65	0.96
Figure L21	Treatment	ACME (white)	0.40	0.80
Figure L21	Treatment	ACME (Black)	0.35	0.80
Figure L21	Enforcement	ACME (white)	0.42	0.80
Figure L21	Enforcement	ACME (Black)	0.40	0.80
Figure L22	Treatment	ACME (woman)	0.92	1.00
Figure L22	Treatment	ACME (man)	0.89	1.00
Figure L22	Enforcement	ACME (woman)	0.97	1.00
Figure L22	Enforcement	ACME (man)	0.88	1.00
Figure M23	Treatment	Race (matching), exposure	0.02	0.36
Figure M23	Treatment	Race (matching), no exposure	0.03	0.46
Figure M23	Treatment	Gender (matching), exposure	0.70	0.97
Figure M23	Treatment	Gender (matching), no exposure	0.39	0.80
Figure M23	Treatment	Context (matching), exposure	0.83	1.00

Figure M23	Treatment	Context (matching), no exposure	0.58	0.90
Figure M23	Enforcement	Race (matching), exposure	0.83	1.00
Figure M23	Enforcement	Race (matching), no exposure	0.04	0.51
Figure M23	Enforcement	Gender (matching), exposure	0.83	1.00
Figure M23	Enforcement	Gender (matching), no exposure	0.08	0.54
Figure M23	Enforcement	Context (matching), exposure	0.45	0.80
Figure M23	Enforcement	Context (matching), no exposure	0.29	0.71
Figure M24	Treatment	Race, exposure	1.00	1.00
Figure M24	Treatment	Race, no exposure	0.53	0.87
Figure M24	Treatment	Race by respondent (Black), exposure	0.07	0.54
Figure M24	Treatment	Race by respondent (Black), no exposure	0.84	1.00
Figure M24	Treatment	Race by respondent (white), exposure	0.25	0.71
Figure M24	Treatment	Race by respondent (white), no exposure	0.11	0.55
Figure M24	Enforcement	Race, exposure	0.20	0.69
Figure M24	Enforcement	Race, no exposure	0.70	0.97
Figure M24	Enforcement	Race by respondent (Black), exposure	0.26	0.71
Figure M24	Enforcement	Race by respondent (Black), no exposure	0.06	0.52
Figure M24	Enforcement	Race by respondent (white), exposure	0.14	0.59
Figure M24	Enforcement	Race by respondent (white), no exposure	0.50	0.86
Figure M25	Treatment	Gender, exposure	0.62	0.94
Figure M25	Treatment	Gender, no exposure	0.53	0.87
Figure M25	Treatment	Gender by respondent (man), exposure	0.56	0.88
Figure M25	Treatment	Gender by respondent (man), no exposure	0.87	1.00
Figure M25	Treatment	Gender by respondent (woman), exposure	0.89	1.00
Figure M25	Treatment	Gender by respondent (woman), no exposure	0.29	0.71
Figure M25	Enforcement	Gender, exposure	0.09	0.55
Figure M25	Enforcement	Gender, no exposure	0.94	1.00
Figure M25	Enforcement	Gender by respondent (man), exposure	0.18	0.68
Figure M25	Enforcement	Gender by respondent (man), no exposure	0.18	0.68
Figure M25	Enforcement	Gender by respondent (woman), exposure	0.29	0.71
Figure M25	Enforcement	Gender by respondent (woman), no exposure	0.15	0.61

Table K-2: Multiple hypothesis testing corrections using q-values

L Mediation Analyses

In this section, we conduct mediation analyses (Imai et al., 2011) to assess the causal mediation of our experimental effects by respondents' perceptions of substance users' blame for their situations. the figures below present the average direct effects (ADEs) of our experimental manipulations, the average causal mediation effects (ACMEs) of perceived blame, and the total (combined) effects. As indicated by the null ACMEs across our different identity manipulations and respondent subgroups, perceptions of blame did not play a causally mediating role in our observed treatment effects.

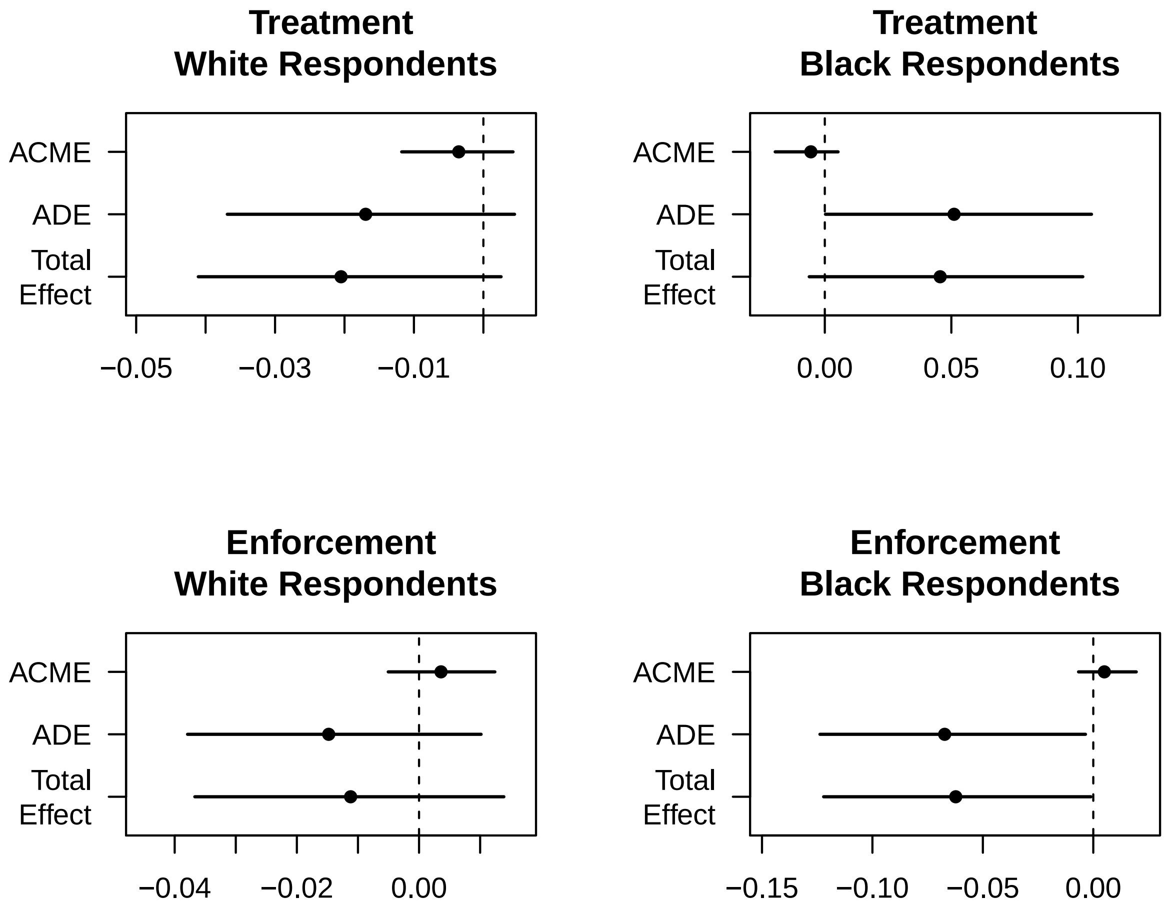

Figure L-21: Treatment effects and confidence intervals by respondent race. Points indicate the difference in levels of support for increasing policy funding between respondents who saw a black substance user profiled and those who saw a white substance user profiled, with 95%-confidence intervals. Total Effect represents a composition of the Average Direct Effect (ADE) and the Average Causal Mediation Effect (ACME).

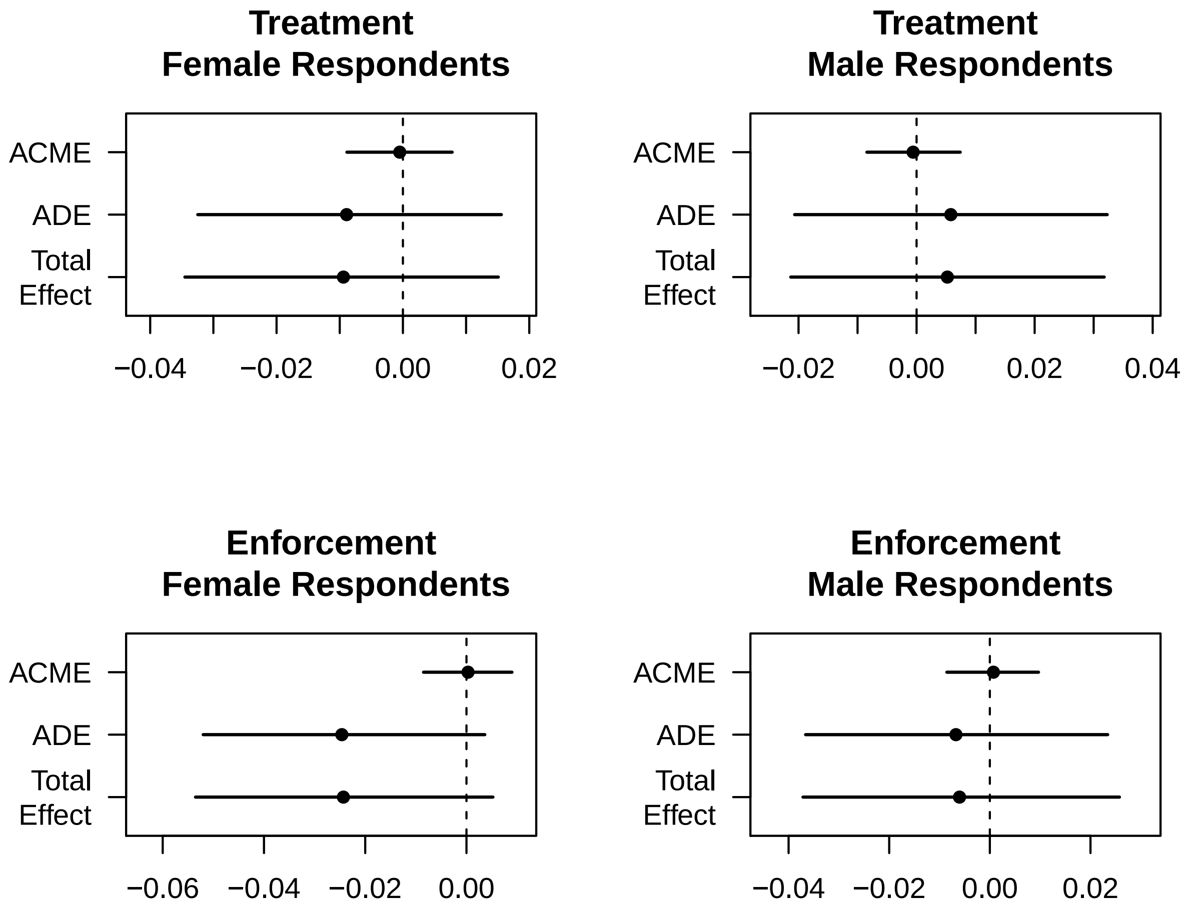

Figure L-22: Treatment effects and confidence intervals by respondent gender. Points indicate the difference in levels of support for increasing policy funding between respondents who saw a female substance user profiled and those who saw a male substance user profiled, with 95%-confidence intervals. Total Effect represents a composition of the Average Direct Effect (ADE) and the Average Causal Mediation Effect (ACME).

M Heterogeneity in the Effects of Group Identity by Personal Exposure

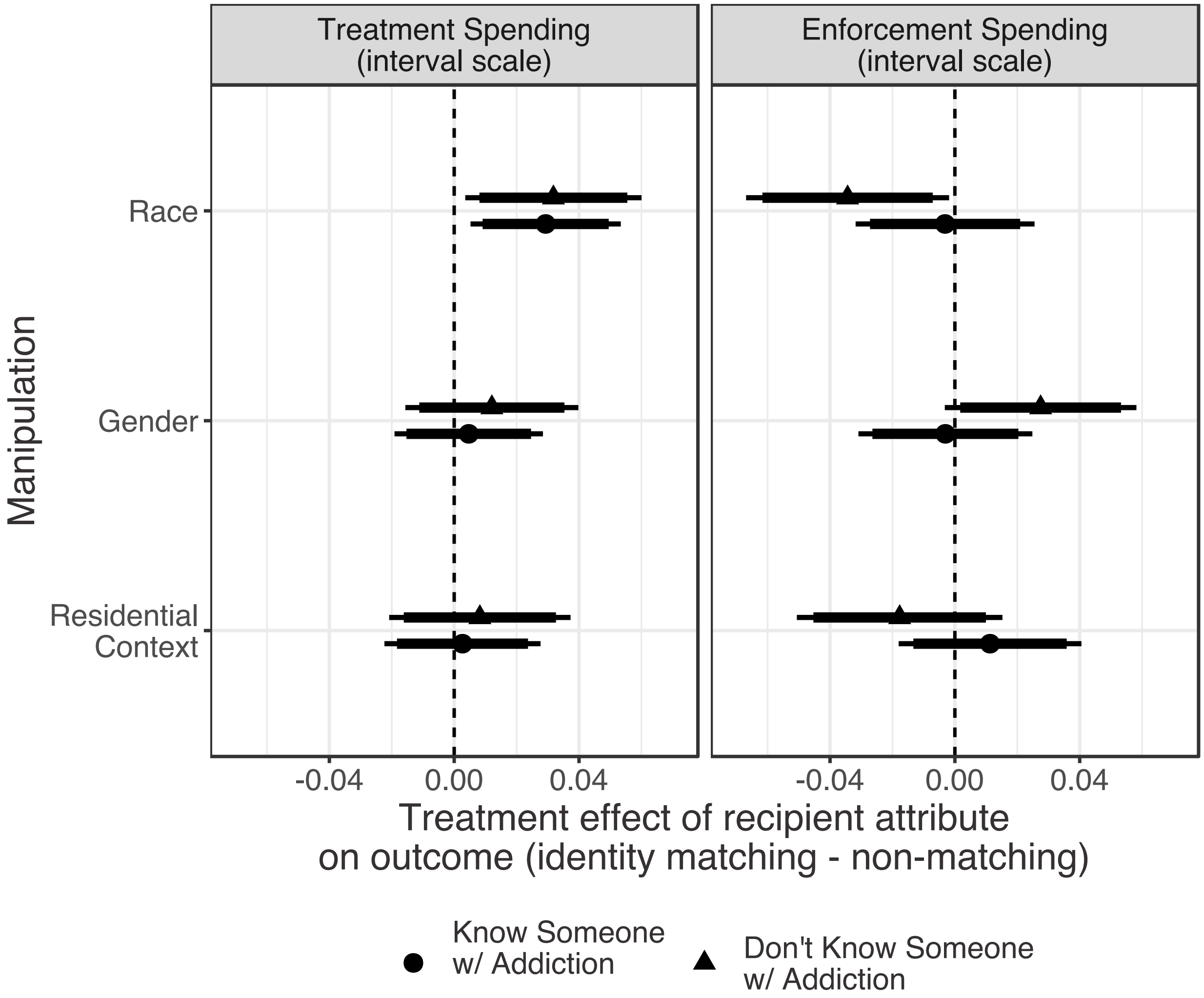

Figure M-23: Treatment effects and confidence intervals for match between respondent characteristic and substance user attributes on unit scale interval outcome. Points indicate the difference in each policy outcome between respondents who matched the individual profiled and those who didn't match them for each of the three identity attributes, with 95%-confidence intervals (thin lines) and 90%-confidence intervals (thick lines). Filled circles indicate treatment effects among respondents who reported knowing someone with opioid addiction issues, and triangles indicate those respondents who reported not knowing anyone struggling with addiction.

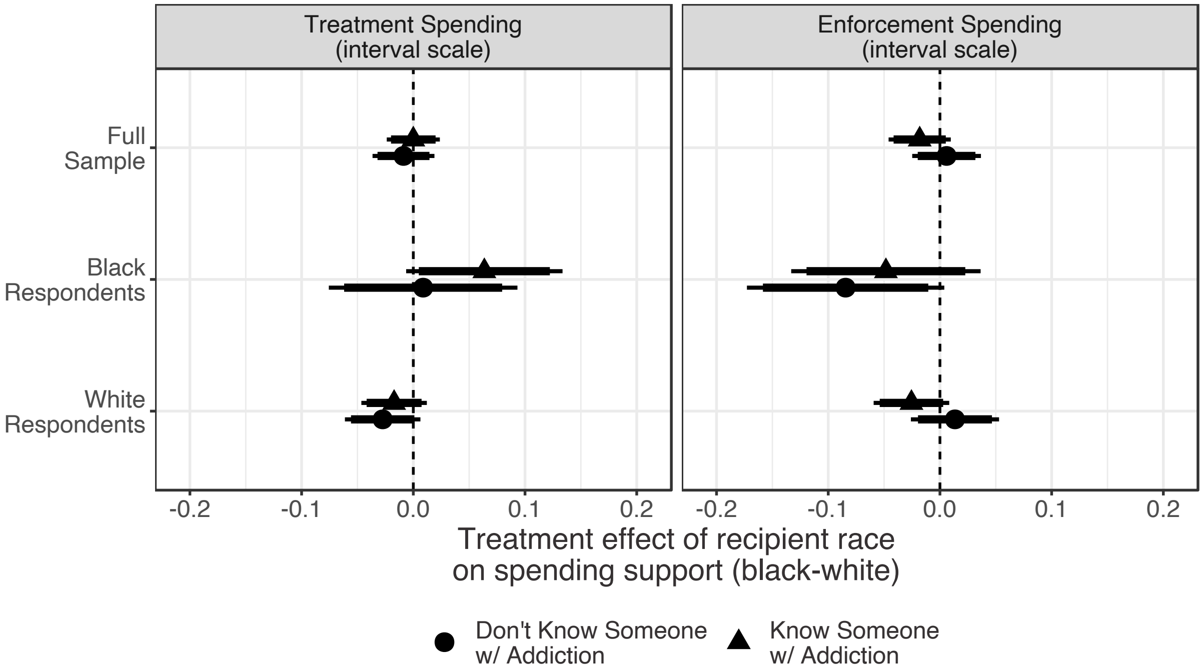

Figure M-24: Treatment effects and confidence intervals by respondent race and personal exposure to addiction. Points indicate the difference in levels of support for increasing policy funding between respondents who saw a black individual profiled and a white individual profiled, with 95%-confidence intervals (thin lines) and 90%-confidence intervals (thick lines). Filled circles indicate treatment effects among respondents who reported knowing someone with opioid addiction issues, and triangles indicate those respondents who reported not knowing anyone struggling with addiction.

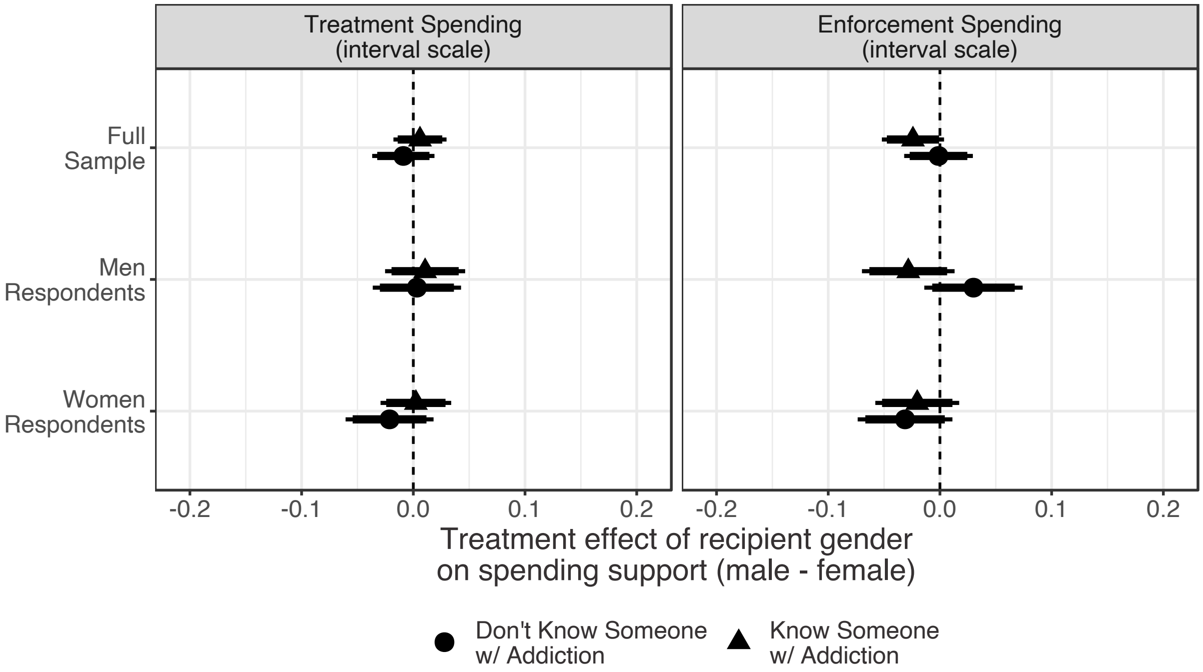

Figure M-25: Treatment effects and confidence intervals by respondent gender. Points indicate the difference in levels of support for increasing policy funding between respondents who saw a male substance user profiled and those who saw a female substance user profiled, with 95%-confidence intervals (thin lines) and 90%-confidence intervals (thick lines). Filled circles indicate treatment effects among respondents who reported knowing someone with opioid addiction issues, and triangles indicate those respondents who reported not knowing anyone struggling with addiction.

N Pre-Analysis Plan

Included below are the hypotheses we test as well as our analytical strategy for testing each hypothesis as pre-registered with EGAP. We note that the hypothesis numbers as noted in the manuscript do not necessarily correspond with the ordering of those hypotheses that we report in this manuscript, which we have adjusted for conceptual clarity. Elements of the pre-analysis plan (the study's theory, experimental design, and survey instrument) that are discussed or included elsewhere in the manuscript are not reproduced below but are included in the PAP filed with EGAP. As noted in the manuscript, our experimental design also included two manipulations unrelated to group identity: how the substance user received treatment for their addiction and how the substance user began using opioids. Results from these manipulations are discussed in other work outside of this paper, and as such we do not display these hypotheses or analytic strategies below.

Hypotheses

Shared Identity

We expect that group identity may sway perceptions of deservingness and subsequent policy support. This leads to:

Hypothesis 1 (H1): *We expect that the race, gender, and location in a rural or non-rural location of policy beneficiaries depicted in a media story will affect support for treatment and punitive policies, operationalized as support for increasing funding for opioid treatment policy and punitive policy. Specifically, for the full sample, we expect a decrease in support for funding after reading about a black policy beneficiary compared to a white policy beneficiary.*

Hypothesis 2 (H2): *We expect that respondents will be more sympathetic to policy beneficiaries who share identities with the respondent – e.g., black respondents will be more sympathetic to black policy beneficiaries depicted in the media, while white respondents will be more sympathetic to white policy beneficiaries. Viewing a profile with a shared identity will increase respondent support for funding treatment policy.*

Policy responses to drug use have traditionally been characterized as emphasizing addiction treatment or punishment (Meier, 1994). To capture both dimensions, we also ask whether respondents support funding for law enforcement to arrest and prosecute drug users. Yet, while drug policy is multi-dimensional, the longitudinal shift away from punishing drug use towards connecting users with treatment suggests these policy attitudes are inversely correlated. This leads to:

Hypothesis 3 (H3): *We expect that any experimental treatment which increases respondent support for funding treatment policy will also decrease respondent support for funding law enforcement to arrest and prosecute drug users.*

The mechanism behind support for both treatment and punishment is the perceived 'deservingness' of the substance users. Capturing the change in sympathy is our outcome variable of blame. A shared identity will decrease agreement with the belief that individuals are to blame for their own addiction. This leads to:

Hypothesis 4 (H4): *We expect the 'blame' outcome variable to negatively correlate with support for funding treatment policy and positively correlate with support for funding punitive policy.*

Personal Exposure

Hypothesis 5 (H5): *We expect that respondents who have personally known someone who has struggled with addiction will express greater support for addiction treatment funding.*

Analytical Strategy

Shared Identity

The following approaches will be used to test each hypothesis:

H1: Two-tailed t-tests of difference in means of treatment funding support between each vignette treatment group (e.g., 'rural') vs. all the others in that category (e.g., 'suburban' and 'urban'), using one treatment group in each category as the baseline category.

H2: Two-tailed t-tests of support for treatment funding on each identity treatment within respondent subgroups detailed below. Second, interact identity treatment effect with an indicator for the respondent's identity subgroup. Third, measure effect of shared identity via omnibus model using new indicator for a shared identity between each respondent to the identity treatment they received.

- For the effect of the race experimental manipulation, subgroups by survey respondent race/ethnicity (black vs. non-Hispanic white).
- For the effect of the gender experimental manipulation, subgroups by respondent gender (male vs. female).
- For the effect of the location experimental manipulation, subgroups by respondent location (rural vs. non-rural, as well as three subgroups matching the manipulated levels of rural, urban, and suburban).

H3: Conduct tests for H1 and H2 using punitive outcome. Repeat additional tests below using punitive outcome.

H4: Conduct tests for H1 and H2 using blame outcome. Repeat additional tests below using blame outcome.

Personal Exposure

H5: Two-tailed t-tests for the difference in means of treatment funding support between respondents based on personal exposure to those with opioid addiction.

References

AmeriSpeak Panel Technical Overview. 2016.

Berinsky, Adam J, Gregory A Huber, and Gabriel S Lenz. 2012. "Evaluating Online Labor Markets for Experimental Research: Amazon.com's Mechanical Turk." *Political Analysis* 20(3): 351–368.

Cramer, Katherine J. 2016. *The Politics of Resentment: Rural Consciousness in Wisconsin and the Rise of Scott Walker*. Chicago: University of Chicago Press.

Gaddis, S Michael. 2017. "How Black are Lakisha and Jamal? Racial Perceptions from Names Used in Correspondence Audit Studies." *Sociological Science* 4: 469–489.

Imai, Kosuke, Luke Keele, Dustin Tingley, and Teppei Yamamoto. 2011. "Unpacking the Black Box of Causality: Learning About Causal Mechanisms from Experimental and Observational Studies." *American Political Science Review* 105(4): 765–789.

Kane, John V, and Jason Barabas. 2019. "No Harm in Checking: Using Factual Manipulation Checks to Assess Attentiveness in Experiments." *American Journal of Political Science* 63(1): 234–249.

Levay, Kevin E, Jeremy Freese, and James N Druckman. 2016. "The Demographic and Political Composition of Mechanical Turk Samples." *Sage Open* 6(1): 1–17.

Meier, Kenneth J. 1994. *The Politics of Sin: Drugs, Alcohol and Public Policy*. Armonk, NY: M.E. Sharpe.

Storey, John D, and Robert Tibshirani. 2003. "Statistical Significance for Genomewide Studies." *Proceedings of the National Academy of Sciences* 100(16): 9440–9445.
